# Recent Advances of Pyridinone in Medicinal Chemistry

**DOI:** 10.3389/fchem.2022.869860

**Published:** 2022-03-23

**Authors:** Shibo Lin, Chun Liu, Xiaotian Zhao, Xiao Han, Xuanhao Li, Yongqin Ye, Zheyu Li

**Affiliations:** ^1^ Department of Pharmacy, Chengdu Second People’s Hospital, Chengdu, China; ^2^ Antibiotics Research and Re-evaluation Key Laboratory of Sichuan Province, School of Pharmacy, Chengdu University, Chengdu, China

**Keywords:** drug-like molecules, medicinal chemistry, pyridinone, biological activity, structure–activity relationship (SAR)

## Abstract

Pyridinones have been adopted as an important block in medicinal chemistry that could serve as hydrogen bond donors and acceptors. With the help of feasible synthesis routes *via* established condensation reactions, the physicochemical properties of such a scaffold could be manipulated by adjustment of polarity, lipophilicity, and hydrogen bonding, and eventually lead to its wide application in fragment-based drug design, biomolecular mimetics, and kinase hinge-binding motifs. In addition, most pyridinone derivatives exhibit various biological activities ranging from antitumor, antimicrobial, anti-inflammatory, and anticoagulant to cardiotonic effects. This review focuses on recent contributions of pyridinone cores to medicinal chemistry, and addresses the structural features and structure–activity relationships (SARs) of each drug-like molecule. These advancements contribute to an in-depth understanding of the potential of this biologically enriched scaffold and expedite the development of its new applications in drug discovery.

## Introduction

**FIGURE 1:**
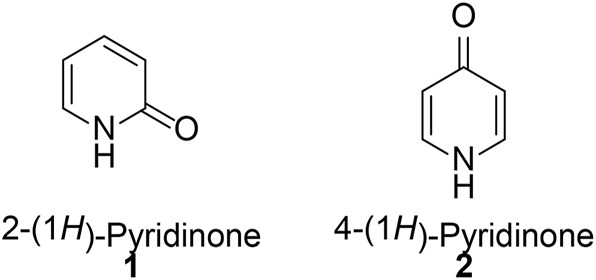
Two regioisomeric forms of pyridinone.

Privileged structure-guided scaffold re-evolution/refining is a primary strategy to identify structurally novel chemotypes by modifying the central core structure and the side chain of the existing active compounds, or to exploit undescribed bioactivites by making full use of readily derivatized motifs with well-established synthetic protocols (Song et al., 2013a; Song et al. 2013b; Song et al. 2014). Pyridinones are an interesting class of six-membered heterocyclic scaffolds with a nitrogen, an oxygen, and five carbon atoms, which have also been employed as bioisosteres for amides, pyridines, pyranones, pyrimidines, pyrazines, and phenol rings. According to the relative position between the nitrogen heteroatom and carbonyl moiety (Katrizky et al., 2010), two isomeric forms including 2- and 4-(1*H*)-pyridinones exist as skeletal components (Figure 1). In addition, a study on isomerization between the pyridinone and the corresponding hydroxypyridine indicates that the former form is favored, especially in physiological conditions.

**FIGURE 2:**
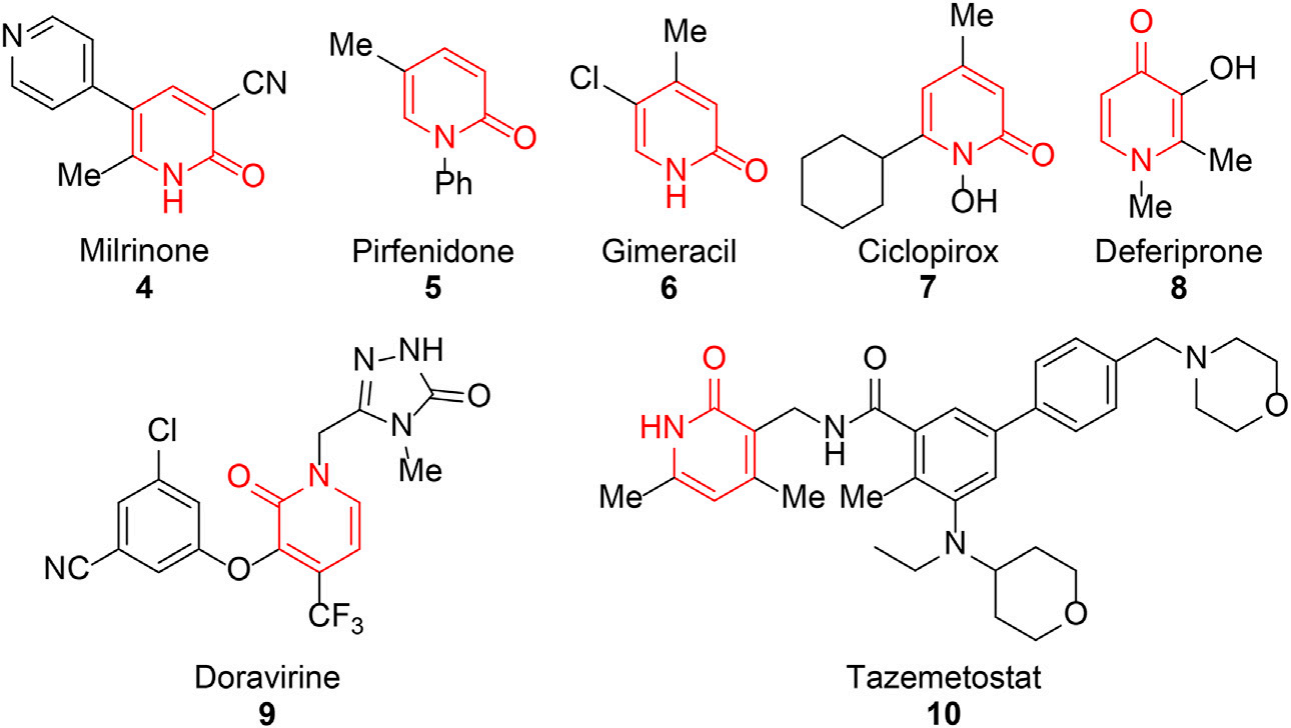
Marketed drugs containing pyridinone.

Despite the small size of pyridinone, it could provide five derivatizable positions possessing four hydrogen bond acceptors and a hydrogen bond donor. Thus, it can form additional interactions with the therapeutic targets, cross the cell membrane easily, and increase the water solubility of molecules. In recent years, pyridinone derivatives have attracted extensive attention due to their versatile pharmaceutical, agricultural, and industrial applications (Zhang and Pike 2021). Protein kinases owning a highly conserved three-dimensional structure in their catalytic domains have become a druggable target class (Santos et al., 2017). Unsubstituted pyridinone could serve as a valid peptide bond isostere and form either a single or multiple H-bond interactions with the kinase hinge region to gain appropriate binding affinity, and have been explored in various kinase programs such as Met kinase, mitogen-activated protein kinase-interacting kinase, orotate phosphoribosyltransferase, monoamine oxidase B, mitogen-activated protein kinase, and Bruton’s tyrosine kinase. Pyridinone-containing compounds show a broad spectrum of pharmacological properties such as antitumor, antimicrobial, anti-inflammatory, antimalarial, antidepressant, and cardiotonic effects. The scaffold has become a hot point in medicinal chemistry, and an increasing number of FDA-approved drugs have reinforced its significance. As depicted in Figure 2, milrinone is a powerful cardiac stimulant, pirfenidone is an agent for idiopathic pulmonary fibrosis (IPF), gimeracil and tazemetostat are considered as antineoplastic drugs, ciclopirox is an antifungal agent, deferiprone is a well-known iron chelator, and doravirine is a valuable anti-HIV drug (Shipley et al., 1996; Niewerth et al., 2003; Cho and Kopp 2010; Harada et al., 2017; Sanchez et al., 2019). It could draw a conclusion that the pattern of substituents on pyridinone exercises considerable influence over the pharmacological properties and its therapeutic applications.

## Preparation of Pyridinone Derivatives

**FIGURE 3:**
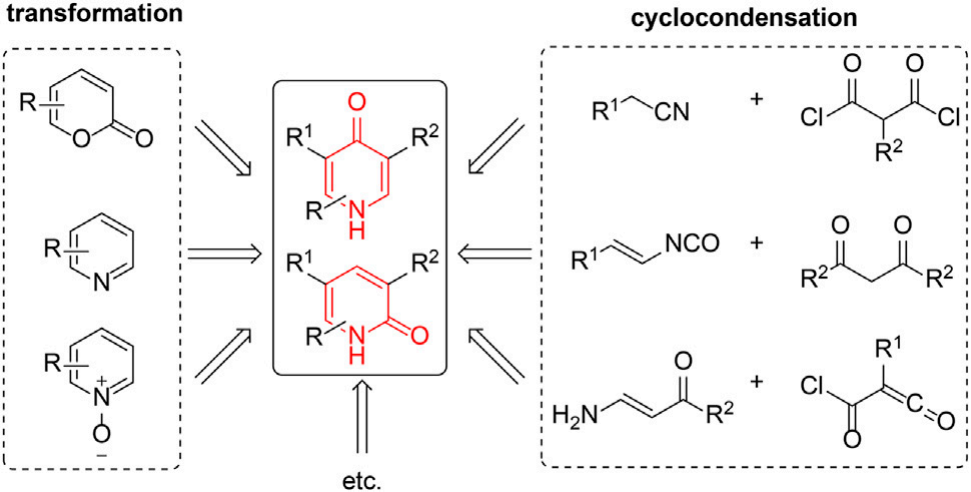
Precursors of pyridinone derivatives.

In the past decades, two main synthetic approaches have been reported to form the ring of pyridinone: one is to synthesize from pyridine or related six-membered ring forms *via* the introduction of carbonyl groups, and the other is by the cyclic condensation of two compounds (Figure 3).

**FIGURE 4:**
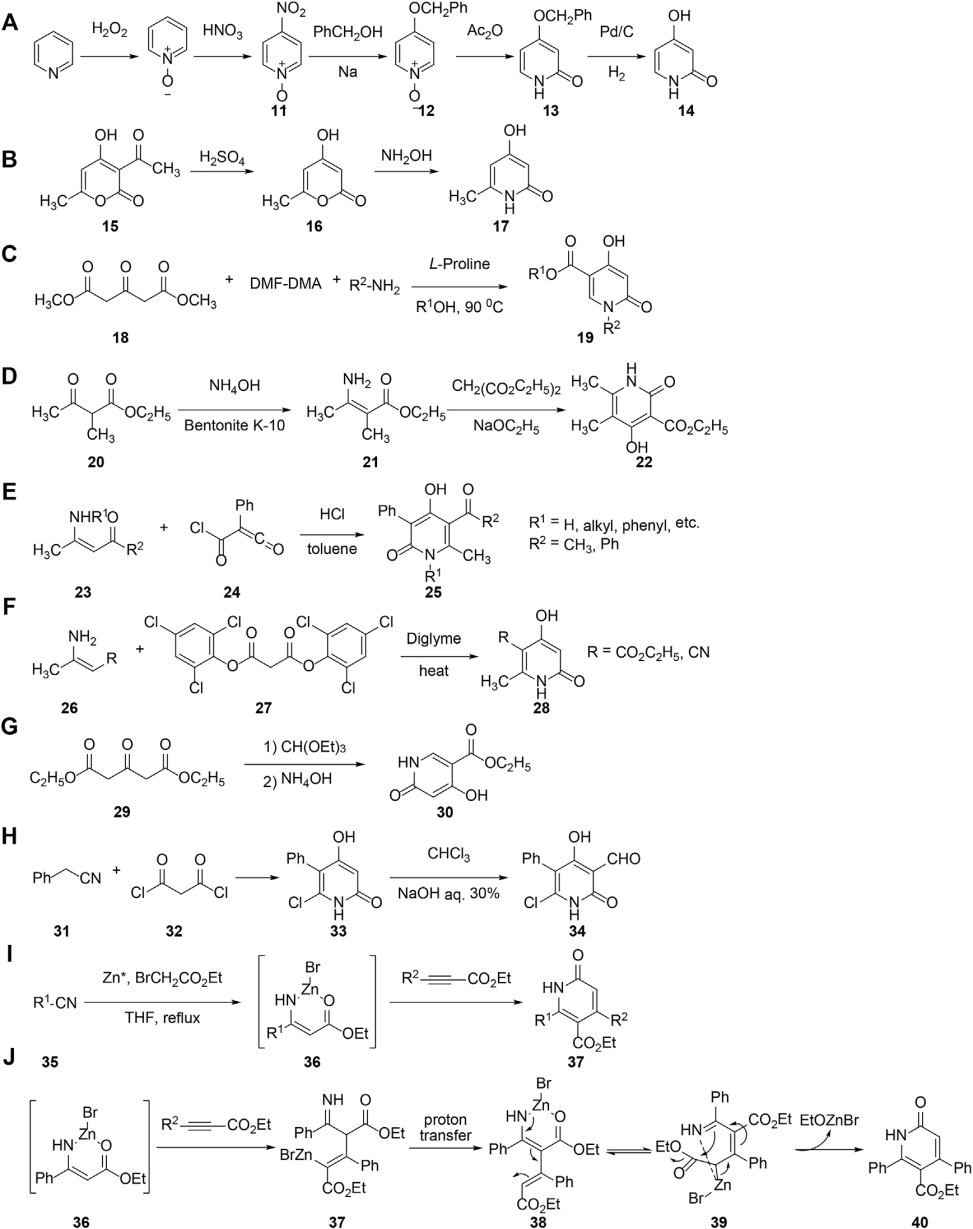
Synthesis of pyridinone with various reactions.

Lin et al. (2003) used pyridine as substrates, and hydrogen peroxide and nitric acid as reagents to get 4-nitropyridine-*N*-oxide, followed by acetylization, and elimination reactions to generate pyridinone (Figure 4A). Another routine for preparing pyridinone (Demuner et al., 2009) is to primarily get deacetylated pyranone and then treat it with ammonia solution (Figure 4B).

Bai et al. (2018) reported a one-pot synthesis of 2-(1*H*)-pyridinone directly from dimethyl 3-oxopentanedioate, *N*,*N*-dimethylformamide dimethyl acetal (DMF-DMA), and primary amine by using *L*-proline as a catalyst. The reaction could proceed efficiently and ecofriendly with broad functional group tolerance (Figure 4C). Ethyl acetoacetate in a medium of aqueous ammonia is readily converted into imino ester (McElroy and DeShong 2003), followed by condensation with diethyl malonate to afford ethyl 4-hydroxy-2-pyridinone-3-carboxylate (Figure 4D). Abaszadeh et al. (2009) reported treatment of (chlorocarbonyl)phenyl ketene with enaminones in boiling toluene afforded 4-hydroxy-3,4,6-trisubstituted-2(1*H*)-pyridinone conveniently and rapidly (Figure 4E). As Guillemont et al. (2009) published, ethyl 3-aminocrotonate was condensed with the activated malonate derivative to afford 4-hydroxy-2-pyridinone conveniently in 75% yield in a single step without added base (Figure 4F). Ethyl 3-oxoglutarate has been reported (Platts et al., 2011) to directly react with triethylorthoformate to obtain ethyl 4-hydroxy-2-pyridinone-5-carboxylate in the presence of ammonia (Figure 4G).

The construction of the pyridinone moiety was carried out first between phenylacetonitrile and malonyl chloride in 56% yield. Then, electrophilic addition of dichlorocarbene species to the intermediate gave the desired product. The moiety can be formylated to afford pyridinone derivatives (Brondel et al., 2006) which are very significant intermediate to further prepare other pyridinones (Figure 4H).

Chun et al. (2009) successively reported a tandem one-pot conversion of nitriles with ethyl bromoacetate in THF to get various 2-pyridinone derivatives *via* the Blaise reaction intermediate, which was demonstrated to be a very efficient and operationally convenient method (Figure 4I). The possible mechanism involves vinyl zinc bromide obtained through Michael addition reaction of the Blaise reaction intermediate, and the propiolate was isomerized to the α-vinylated zinc bromide complex, followed by rearrangement and intramolecular ring-closing reaction to yield the 2-pyridinone structure (Figure 4J).

Despite its importance as a special scaffold in drug research, reviews on the medicinal application of pyridinone-containing derivatives are still rare. Consequently, the following subsections aim to present recent advances in bioactivity of pyridinone scaffolds, discuss the diversity of pharmacological and biological action, and exhibit the selected data to demonstrate structure–activity relationships (SARs) and the proposed mechanisms with their targets.

## Biological Interest of Pyridinone Derivatives

### Anticancer Activity

Cancer, known as malignant tumor, is a multifactor-causing disease (Atkins and Gershell 2002). Tumorigenesis is accompanied by many physiological and biochemical phenomena, for example, the uncontrolled cell growth and apoptosis, tumor angiogenesis, lower pH value, hypoxia, and metastasis (Jemal et al., 2013).

Novel pyridinone-containing molecules have attracted considerable attention for their broad-spectrum of antiproliferative activity against various human tumor cell lines. The useful scaffold can specifically arrest some targets including protein tyrosine kinases, Met kinase, histone deacetylase (HDAC), isocitrate dehydrogenase (IDH), cyclin, mitogen-activated protein kinase (MAPK), and ribonucleotide reductase. Accordingly, we classified the published derivatives into the following types based on different targets to control or prevent cancer.

#### Kinase Inhibitors

**FIGURE 5:**
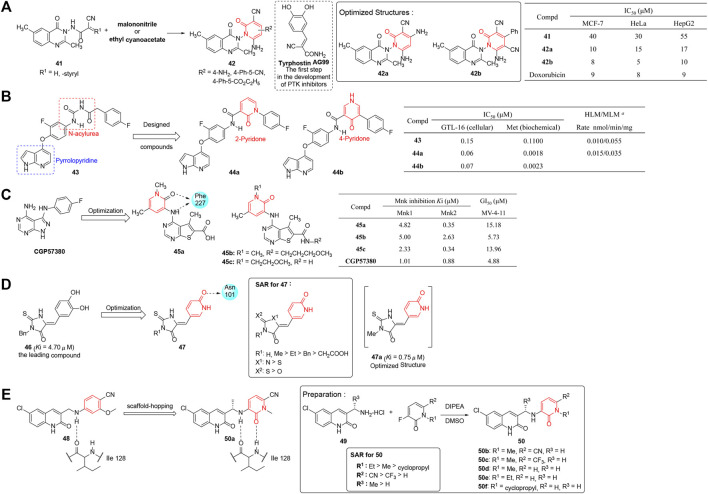
**(A)** Quinazoline-conjugated pyridinones were designed in the discovery of new PTK inhibitors (Alafeefy et al., 2012). **(B)** Pyrrolopyridine-pyridinone-based inhibitors of Met kinase (Kim et al., 2008). ^*a*^ Compounds at 3 μM incubated in 10 mg of protein (human (HLM) and mouse (MLM) liver microsome) for 10 min. **(C)** Pyridinone–thienopyrimidine derivatives were designed as potent and highly selective MNK inhibitor (Yu et al., 2015). **(D)** Synthesis of pyridinone–thiohydantoin derivatives and their SARs against IDH1 (R132H). **(E)** Synthesis and SAR of pyridinone-quinolinones as IDH1 (R132H) inhibitors.

Protein phosphorylation (Le Quesne et al., 2010) is a significant signal transduction pathway modulated by protein kinases (PKs) and phosphatases which is essential for living cells. PKs are regulatory enzymes that transfer the γ phosphate from ATP to the amino acid residues of protein substrate, while phosphatases perform the opposite process (Bryan and Rajapaksa 2018). Alafeefy et al. (2012) reported a series of pyridinone–quinazoline derivatives (**42**) as cytotoxic agents targeting protein tyrosine kinases (PTK). The analogs were synthesized by condensation of substituted quinazoline and malononitrile or ethyl cyanoacetate, the former of which were prepared from 2-amino-5-methylbenzoic acid. The synthesized compounds were screened for preliminary anticancer activity using three tumor cell lines, MCF-7, HeLa, and HepG2 *in vitro*. The results in Figure 5A demonstrated that all the pyridinone–quinazoline derivatives displayed >70% inhibition with improved IC_50_ values ranging from 9 to 15 μM and displayed suitable drug-like characteristics according to Lipinski’s rule of five. Most of the active compounds had a considerable number of hydrogen bond donors and acceptor groups comparable to model tyrphostin **AG99**. Among them, **42a** and **42b** were as potent as doxorubicin in anticancer activity and exhibited good bioavailability. Such observation rendered the series of pyridinone–quinazoline analogs deserving deeper research and development.

Kim et al. (2008) synthesized a series of pyridinone-pyrrolopyridine-based inhibitors of Met kinase. With **43** as the lead compound, the pyrrolopyridine moiety was retained as the basic skeletal structure, and modification was mainly focused on replacing the acylurea moiety by pyridinone ring acting as a conformationally constraint role and increasing the carbonyl oxygen hydrogen bonds with the backbone NH of Asp1222 (Figure 5B). In this study, twenty-five derivatives were synthesized and evaluated for their antiproliferative activities by using the GTL-16 gastric carcinoma cell line and Met kinase assay. The result showed that compounds **44a** and **44b** enhanced the potency markedly (IC_50_ = 0.06 and 0.07 μM, respectively) and possessed excellent metabolic and pharmacokinetic parameters. The compounds also demonstrated good *in vivo* efficacy in the xenograft model. In addition, X-ray crystallography demonstrated **44a** bound to the ATP-binding pocket of the Met kinase domain.

Yu et al. (2015) reported various pyridinone–thienopyrimidine derivatives which were designed based on the ChemBridge database targeting mitogen-activated protein kinase (MAPK)-interacting kinases (MNKs) whose phosphorylation process played a key role in oncogenic transformation. Compared with **CGP57380**, a known MNK inhibitor with unconquered kinase selectivity, serving as a positive reference substance, most compounds displayed moderate to potent MNK inhibitory activity with high selectivity toward MNK2. In particular, three compounds, **45a**, **45b**, and **45c**, exhibited high activity in both MNK inhibition potency and antiproliferative effects (Figure 5C). Cellular mechanistic studies revealed the compounds were capable of downregulating the phosphorylated eIF4E, Mcl-1, and cyclin D1, and causing PARP cleavage. Interestingly, according to the docking with ATP binding pocket of MNK2, this fraction of pyridinone could generate multiple hydrophobic interactions and hydrogen bonds with Lys113, Arg125, and Phe159. This suggested the exploration of such structural diversity would provide potent and highly selective MNK inhibitors.

#### Isocitrate Dehydrogenase Inhibitors

Isocitrate dehydrogenase (IDH) is one of the key enzymes in the tricarboxylic acid (TCA) cycle which is involved in catalyzing oxidative decarboxylation of isocitric acid to α-ketoglutaric acid. Recent research (Yan et al., 2009; Paschka et al., 2010; Borger et al., 2012) revealed that frequent hematologic and somatic cell variation of IDH1 have been related to certain cancers, including myelodysplastic syndromes (MDS), gliomas (grade II and III), acute myeloid leukemia (AML), intrahepatic cholangiocarcinoma, and some other sarcomas. Therefore, inhibitors of mutant IDH1 are counted on to provide a therapeutic benefit in related cancers.

Based on the modification of 3-benzyl-5-(3,4-dihydroxybenzylidene)-2-thiohydantoin (**46**, *K*_i_ = 4.7 μM) which was published previously (Liu et al., 2014), Wu et al. (2015) prepared a series of pyridinone–thiohydantoin derivatives as inhibitors of mutant IDH1. The inhibitory value (*K*_i_) against mutant IDH1 (R132H) was tested, and results revealed that most compounds possessed improved inhibitory activities (*K*_i_ = 0.42–9.2 μM). In addition, most inhibitors could reduce the concentrations of D2HG, decrease hypermethyled phenotype of histones, and suppress the self-renewal ability of stem-like cancer cells in cell biology studies. The partial SAR showed that 2-thiohydantoin ring was the most favorable core, and a small substituent was more favored at the 3-position (Figure 5D). Remarkably, X-ray crystallography revealed **47a** binded well to IDH1, among which the pyridinone moiety had favorable interactions with Asn101 and Gly97. In addition, studies showed a strong H-bond acceptor (pyridinone) instead of an H-bond donor (hydroxylbenzenyl) was essential for good inhibitory activity. This provided a platform for the design of structure-based inhibitors targeting IDH1 mutated cancers in more depth.

Caravella et al. (2020) synthesized a series of pyridinone quinolinones as mutant-specific inhibitors of IDH1. As shown in Figure 5E, the derivatives were designed on the basis of a previously reported ligand (**48**), by using a scaffold hopping approach. In consideration of the crystal structure of **48** in complex with IDH1 (R132H), which led to suboptimal potency and low solubility, the phenyl ring was changed to pyridinone to improve properties by increasing a hydrogen bond acceptor. In that study, crystal structures of **50** were synthesized through nucleophile substitution reaction, and further evaluated for their IDH1 (R132H) inhibition activity and solubility. Based on structure optimization and overall SAR that showed substitutions at position 7 of the quinolinone ring could improve potency, compound **50a** was discovered as the clinical candidate for its potent and specific inhibition of mIDH1 in hematologic malignancies, solid tumors, and gliomas (NCT02719574 and NCT03684811). In particular, **50a** possesses perfect ADME/PK properties and lowers 2-hydroxyglutarate levels in *in vivo* IDH1 xenograft models.

#### DNA or Protein Inhibitors

Organometallic complexes (Jamieson and Lippard 1999; Louie and Meade 1999; Hambley 2007; Meggers 2009; Dörr and Meggers 2014) are well-known and clinically proven chemotherapeutic agents, of which the representative drugs are cisplatin, carboplatin, and oxaliplatin. On entry into cells, the agents will react with DNA or protein and realize their antitumor properties (Hartinger and Dyson 2009). In recent years, a number of metal complexes were investigated for treatment of a broader range of tumors, with less side effects and higher therapeutic index.

**FIGURE 6:**
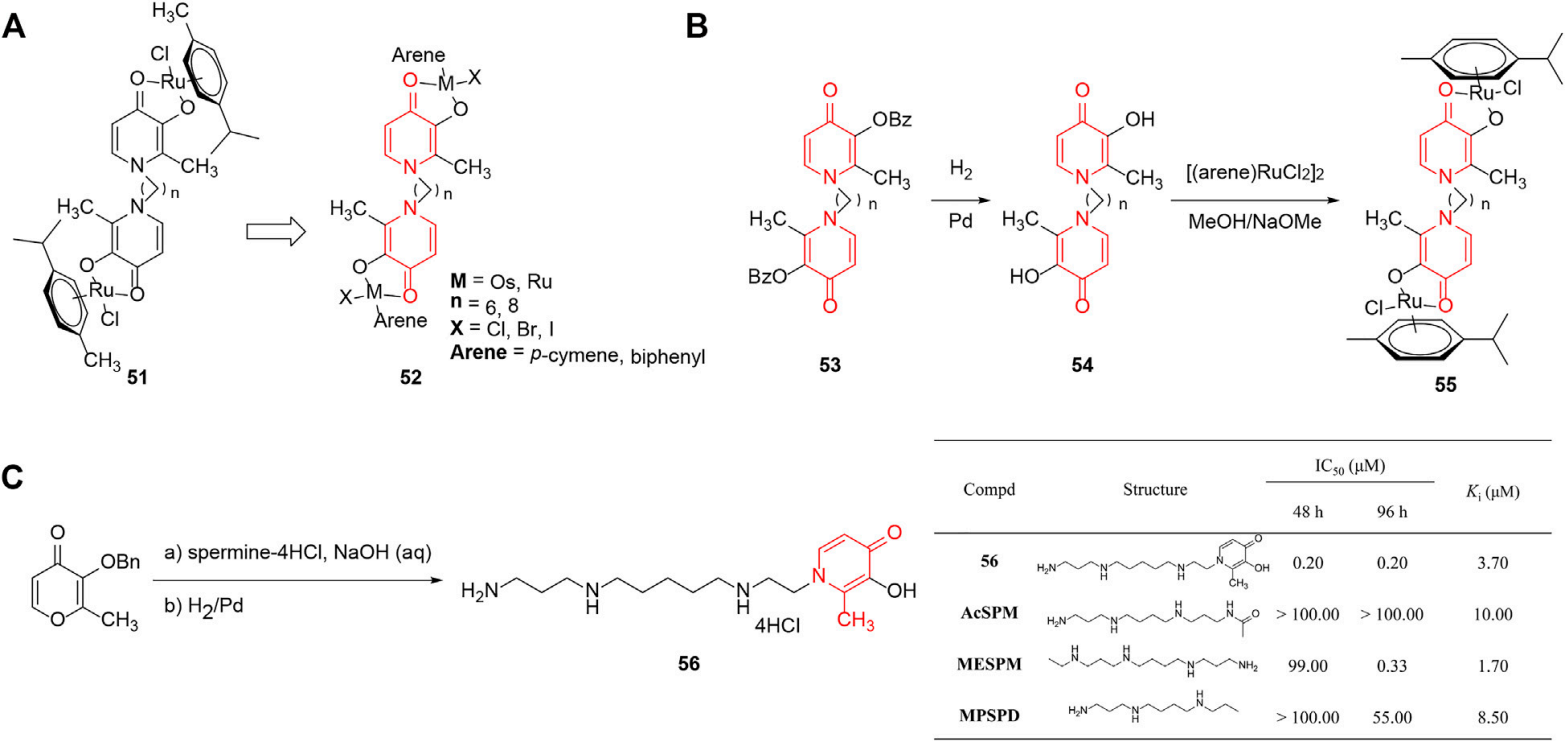
**(A)** SAR of ruthenium(II)–arene complexes linked by pyridinone-based ligands. **(B)** Synthesis and SAR of di-nuclear pyridinone-derived ruthenium complexes. **(C)** Cell growth inhibition and competition transport for pyridinone-containing polyamines (Bergeron et al., 2003).

Mendoza-Ferri et al. (2009a) discovered a number of ruthenium(II)–arene complexes which were derived from **51** to investigate the inhibitory action of the metal centers, the spacer length, the halide ligand, and the arene ligand on tumor cells (Mendoza-Ferri et al., 2009b). The analogs were linked by pyridinone-based ligands and the activities were measured using the MTT assay against the colon and ovarian cancer cell lines. The SAR summarized in Figure 6A demonstrated that the number and nature of metal centers significantly influenced the *in vitro* antineoplastic potency, due to the binuclear complex with Ru possessing increased solubility and lipophilicity. However, the type of halide made little difference owing to predominant formation of the same aquation product. Notably, in addition to lipophilicity, the spacer length relevant for the capability of hitting biomolecular targets also caused a pronounced role in the mode of action and altered the antitumor activity to a meaningful extent.

The same group (Mendoza-Ferri et al., 2009a) reported that di-nuclear pyridinone-derived ruthenium complexes which displayed affinity toward transferrin and DNA were potential anticancer drugs. The complexes were generated by the reaction between bis(3-hydroxy-2-methyl-4-pyridinon-1-yl) alkanes and [(arene)RuCl_2_]_2_ in good yields, and were further evaluated for cytotoxicity against several lines. The data showed that chain length influenced the *in vitro* activity seriously, which could be explained by the association with lipophilicity (Figure 6B). Moreover, the most cytotoxic compound **55a** exhibited the same level of IC_50_ values with cisplatin and oxoplatin, and showed no cross-resistance to oxoplatin. Bioanalytical characterization of diaqua species hydrolyzed by the ruthenium complexes exhibited affinity toward transferrin and DNA, suggesting that both proteins and nucleotides were potential targets.

Polyamines could act as vectors that deliver the bidentate chelator into the cell and serve as antineoplastic devices (Yan et al., 2005). Bergeron et al. (2003) designed 1-(12-amino-4,9-diazadodecyl)-2-methyl-3-hydroxy-4(1*H*)-pyridinone (**56**) and synthesized the conjugate by using 3-*O*-benzylmaltol and spermine as raw materials. As depicted in Figure 6C, compound **56** exhibited much better inhibitory activity (at least 230 times) than several other representative polyamines against leukemia cell, and was concentrated in the cell by more than 1900-fold against a gradient due to its efficient diffusion across the membranes. A subsequent study discovered that the chelators forming a complex with Fe(III) could inhibit tumor growth significantly through suppression of ornithine decarboxylase (ODC) and S-adenosylmethionine decarboxylase (AdoMetDC) and upregulating spermidine-spermine *N* ^1^-acetyltransferase (SSAT). In addition, the conjugates could serve as efficient vectors for the intracellular transportation of other metal chelators, and this supplied an attractive system for further exploration of the impact of polyamine delivery.

#### BRD9 Inhibitors

**FIGURE 7:**
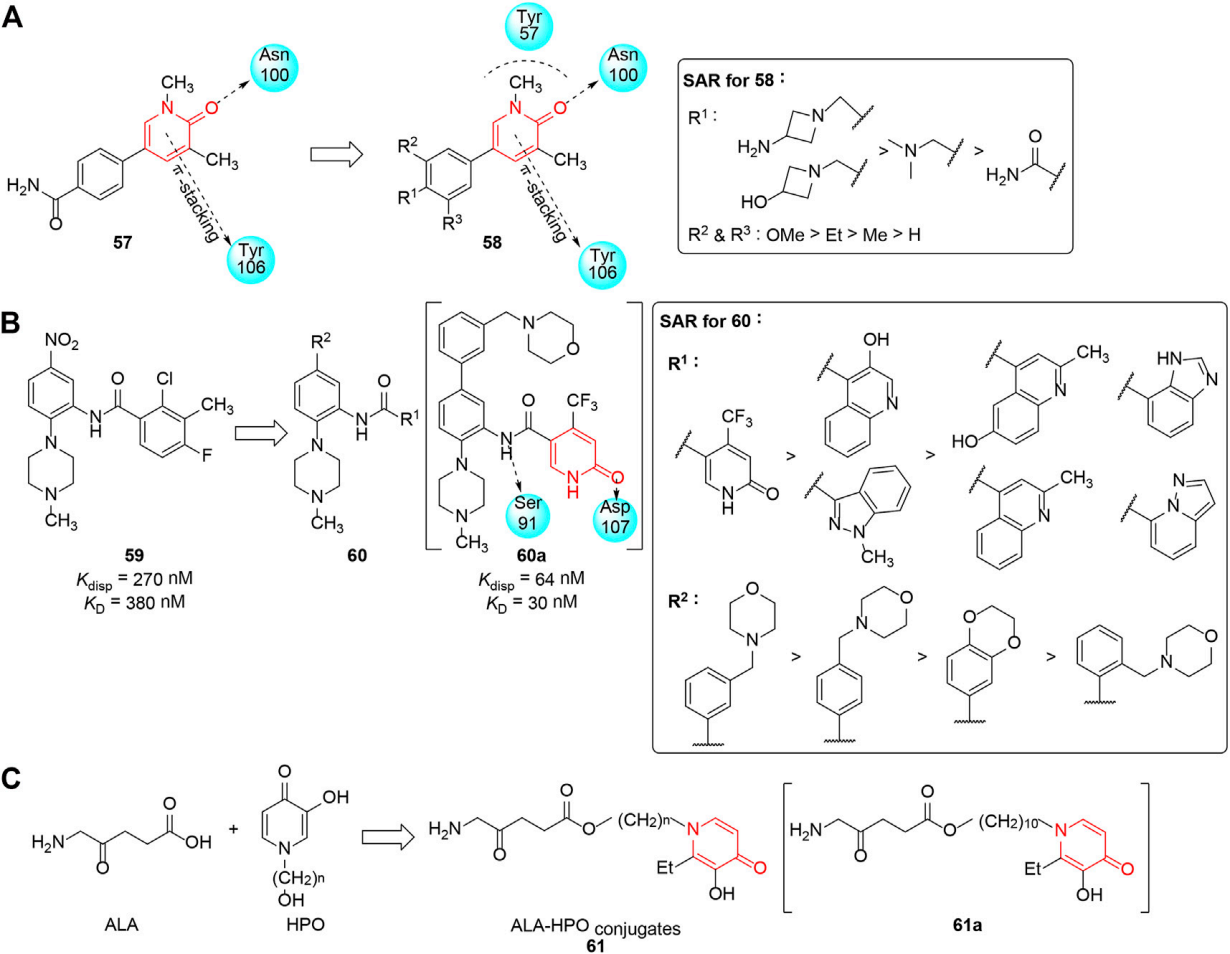
**(A)** SAR of the aromatic pyridinones as BRD9 inhibitors. **(B)** Pyridinones with inhibitory activity against WDR5–MLL1 interaction. **(C)** ALA-HPO conjugates as phototoxic agents.

The mammalian switch/sucrose non-fermentable (SWI/SNF) complex (Zeng and Zhou 2002; Hewings et al., 2012; Fedorov et al., 2015) is associated with chromatin remodeling and is highlighted as a potential anticancer target because the inhibition of remodeling complexes results in the arrest of leukemic cells in G1 and their differentiation. Bromodomain-containing protein 9 (BRD9), a SWI/SNF subunit, participates in the proliferation of acute myeloid leukemia (AML) cells. Therefore, BRD9 is proposed to be a new target for therapeutic strategies for AML (Vidler et al., 2012; Hohmann et al., 2016). In this regard, Martin et al. (2016) selected pyridinone scaffold compounds through Glide docking in high-concentration screening (HiCoS) library and filtering on the basis of molecular weight and lipophilicity. **57** was identified as an initial hit compound which could inhibit BDR9 with IC_50_ of 9.4 μM. Docking analysis of **57** in BDR9 elucidated the pyridinone moiety made important interactions with Asn100, Tyr57, and Tyr106, and the phenyl core had a π-interaction with Ile53. In order to improve the efficacy of **57**, substituents were added to the phenyl group, and most of the derivatives showed potent inhibitory activity at nanomolar concentrations. The study revealed that the additional dimethoxyphenyl linker induced a favorable interaction with Phe47 and realized optimal T-stacking with Phe44, and the partial SAR is shown in Figure 7A. Being available to the scientific community as potent, selective, cell-permeable, and non-cytotoxic BRD9 inhibitors, such molecular probes provided a useful tool to broaden the field of chromatin regulators in medical oncology.

#### WDR5–MLL1 Interaction Inhibitors

Mixed-lineage leukemia 1 (MLL1) is a histone H3K4 methyltransferase, and plays an important role in gene regulation, development, and differentiation (Karatas et al., 2013). As an essential component of MLL1, WD repeat-containing protein 5 (WDR5) is critical for its stability and activity through combination with histone H3, and its chromosomal translocation has a close connection with mixed lineage leukemia (Wysocka et al., 2005; Ang et al., 2011; Bailey et al., 2015). Thus, inhibition of WDR5 might be a novel oncological therapeutic strategy. Getlik et al. (2016) developed N-phenyl-6-oxo-1,6-dihydropyridine-3-carboxamides as new class of WDR5 antagonists, which were designed on the basis of a previous discovery of inhibitor **59** by the same group (Chen et al., 2021). Two series of heteroaryl substituents at the C-5 position of the phenyl ring and C-1 position of amide were synthesized, respectively, and the WDR5-MLL1 *K*_disp_ values of optimized compounds revealed suitable moieties such as pyridinone could significantly improve the overall properties in addition to potency. Functionally, the potency of compounds was assessed using fluorescein-labeled 9-Ala-FAM peptide, and a partial SAR revealed that the introduction of morpholine-substituted phenyl rings at C-5 was crucial for the improvement of binding affinity and solubility (Figure 7B). In particular, compound **60a** exhibited excellent *in vitro* efficacy (*K*_disp_ = 64 nM) and good pharmacokinetic profiles (*via* IP administration); it also decreased the survival time of human AML cells with N-terminal C/EBPα mutations (IC_50_ ≈ 5 μM). This allowed **60a** to not only serve as a potent and selective chemical probe for disclosing the interaction between WDR5 and MLL1 but also be a candidate for the treatment of MLL1-driven acute myeloid leukemias.

#### Phototoxic Agents

Photodynamic therapy (PDT) is an effective targeted therapy (Vrouenraets et al., 2003; Agostinis et al., 2011) that utilizes photosensitizer, light, and oxygen to treat malignant and non-malignant tumor. As a traditional photosensitizer, the clinical potential of 5-aminolaevulinic acid (ALA), a precursor of protoporphyrin IX (PpIX), is hindered by its relatively poor bioavailability due to the hydrophilic nature (Brackett and Gollnick 2011). Considering the excellent cell membrane permeability of 3-hydroxypyridin-4-one chelators (HPOs), Battah et al. (2017) conjugated ALA with HPO iron chelators *via* ester bonds, which would hydrolyze enzymatically under cells’ internal environment and release the two agents following cellular uptake. Compared with the administration of ALA alone, the newly synthesized ALA-HPO conjugate **61** significantly increased phototoxicity and elevated phototherapeutic metabolite formation in all tested cell lines, especially following exposure to light (Figure 7C). Considering the elevation of cellular PpIX levels, compound **61a**, containing a linker of (CH_2_)_10_, was found to be the most phototoxic compound with comparable efficacy to the ALA hexyl ester. Further investigation demonstrated that the main mechanism of ALA-HPO conjugates adopt a passive diffusion pathway, while the uptake of ALA was an active transportation coupled with ATP. This study demonstrates that ALA-HPOs provided a promising therapy for many types of cancer.

## Antiviral Activity

In the late years, diseases induced by viral infection have become increasingly prevalent and widely spread in the world. However, many disadvantages such as multidrug resistance, severe adverse effects, and high cost of some antiviral drugs have caused great disturbance to patients (Drew 2010; Bobrowski et al., 2020). These restrictions have emphasized the urgent demand to develop new drugs for antiviral treatments. A growing body of research has focused on pyridinone-containing compounds, which are proven to be a significant class of leading scaffolds to discover novel effective therapeutics (Zhou et al., 2018).

### Influenza Endonuclease Inhibitors

**FIGURE 8:**
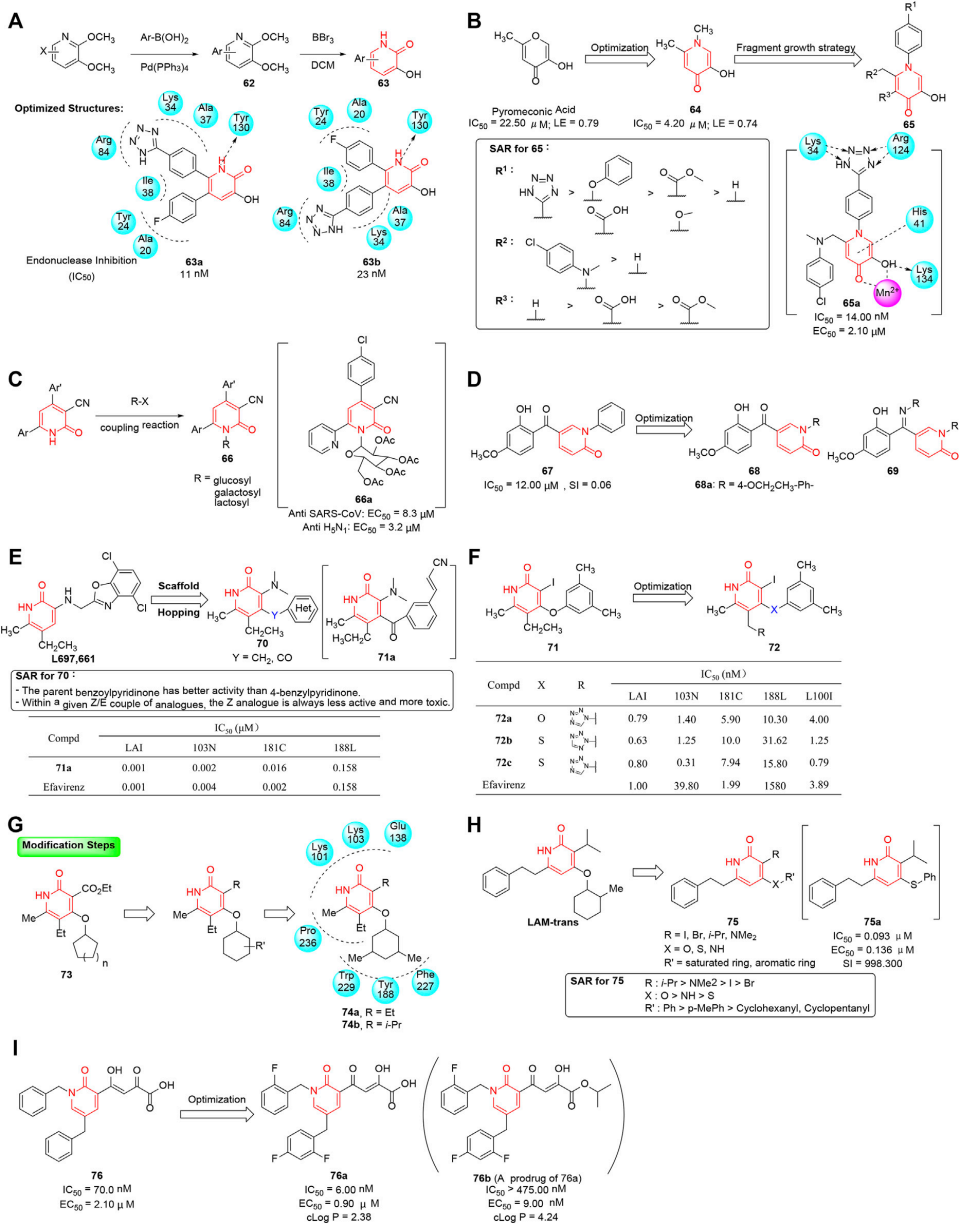
**(A)** Synthesis of phenyl substituted 3-hydroxypyridin-2(1*H*)-ones as endonuclease inhibitors. **(B)** From hit to lead for inhibitors of influenza PA endonuclease. **(C)** Antiviral activity of 2-ONN-based nucleoside analogs. **(D)** 2-pyridinone derivatives as inhibitors against HBV DNA replication and hepatitis B e-antigen (HBeAg) separately. **(E)** Design of pyridin-2(1*H*)-ones as NNRTIs by using a scaffold hopping approach (Benjahad et al., 2004). **(F)** Biological activity of IOPY and ISPY analogs (Benjahad et al., 2007). **(G)** Optimization of pyridinone-based NNRTIs. **(H)** SAR of pyridin-2(1*H*)-ones as inhibitors of HIV reverse transcriptase. **(I)** Optimization of 1,5-dibenzyl-2-pyridinones as InSTIs.

Polymerase acidic (PA) polypeptide chain which is an essential part of the heterotrimeric influenza RNA-dependent RNA polymerase (RdRP) plays a major role in the replication of influenza viruses (Xiao et al., 2006; Dias et al., 2009; Venkataraman et al., 2018). Thus, the endonuclease of the PA subunit becomes a promising target for the treatment of influenza infection. Parhi et al. utilized high-throughput X-ray crystallography fragment screening to discover effective endonuclease inhibitors as potential influenza drugs, and 5-chloro-3-hydroxypyridin-2(1*H*)-one was identified as the chelating ligand (Parhi et al., 2013). A series of such derivatives were then designed and synthesized in two steps, where halogenated 2,3-dimethoxypyridines were first treated with arylboronic acid under Suzuki-coupling conditions, and then further demethylation was performed to yield the desired aryl substituted 3-hydroxypyridin-2(1*H*)-ones (Figure 8A). Their inhibition ability for influenza A endonuclease was evaluated through a high-throughput fluorescence assay, and corresponding SAR indicated the substitutions of diphenyl groups at both the adjacent 5- and 6-positions induced a significant enhancement on potency. Interestingly, **63a** and **63b**, the two more potent analogs in this series, showed dramatically different binding modes that made interactions with the two active site metal ions (M1 and M2) using only two chelating groups, offering a reliable basis on developing a new class of anti-influenza drugs.

Further crystallographic and biochemical studies have demonstrated that a dinuclear metal active site which employed Mn^2+^ and Mg^2+^ cations existed in the endonuclease functional site of the PA subunit. Using metal-binding pharmacophore (MBP) library, the efficient fragments targeting the metal active spot could be found more time- and cost-effectively (Jacobsen et al., 2011), yielding a number of active hits and ultimately leading to the discovery of potent lead molecules. In this regard, Credille et al. (2016) screened pyromeconic acid as a moderate inhibitor of endonuclease activity (IC_50_ = 22.50 μM; ligand efficiency = 0.79). Further optimization was conducted by conversion of the pyrone ring to an N-methylpyridinone (**64**) resulting in a marked increase in potency (IC_50_ = 4.20 μM, ligand efficiency = 0.74), due to more aromatic character and greater electron density on the donating oxygen atoms. By applying “fragment growth” and “fragment merging” strategies, substituents were added at 4′-position of the N-inserted phenyl ring or 6-position of pyridinone and resulted in a marked increase in potency (Figure 8B). In particular, compound **65a** (IC_50_ = 14.00 nM, EC_50_ = 2.10 μM) showed 4- to 5-fold more potent than **L-742,001** (EC_50_ = 9.00 μM) which was one of the most potent inhibitors of influenza PA endonuclease reported in the viral challenge assay (Stevaert et al., 2013). Docking analysis of **65a** in the PA subunit active site showed that 6-position phenylaminomethyl moiety could make an interaction with hydrophobic pocket, and the N-phenyltetrazole moiety was able to form a hydrogen bond with Arg124 and Lys34.

Nucleoside analogs are well-known potent antiviral agents (De Clercq 2004). Abou-Elkhair et al. (2014) reported a scaffold of 2-oxonicotinonitrile (2-ONN)-based acetylated nucleosides. The hybrids were designed on the basis of many published biologically active 2-ONN derivatives. The 2-ONN glucosides were synthesized by coupling 2-ONNs with bromosugars and evaluating the *in vitro* antivirus activity against influenza A virus (H_5_N_1_ and H_3_N_4_ serotype) and severe acute respiratory syndrome coronavirus (SARS-CoV). The selected acetylated 2-ONN glucoside **66a** showed best antiviral activity (Figure 8C).

### Hepatitis B Virus Inhibitors

Hepatitis B virus (HBV) can cause both acute and chronic infections, leading to liver damage, cirrhosis, and hepatocellular carcinoma (HCC) with high mortality (Robinson 1994; Bhattacharya and Thio 2010). Among all DNA viruses, HBV is the most variable one because of its high mutation rate, replicative capability, and virion production. α-interferon is the major therapeutic option for the treatment of HBV infection till now, but the low success rate and serious side effects limited its application (Lavanchy 2004). Unlike all reported anti-HBV agents, 5-(2-hydroxy-4-methoxybenzoyl)-1-phenylpyridin-2(1*H*)-one (**67**) was remarkable for its unique chemical scaffold and was screened as a modest anti-HBV agent in an in-house library by Lv et al. (2010) *.* With **67** as the starting point, a series of 2-pyridinone analogs were synthesized and evaluated for inhibitory activity against HBV-DNA replication, and preliminary SARs showed that the *N*-aryl derivatives exhibited better anti-HBV activity than the *N*-alkyl derivatives (Figure 8D). Among them, compound **68a** exhibited the best inhibitory activity against HBV DNA replication (IC_50_ = 0.12 μM) and high selectivity (selectivity index: CC_50_/IC_50_ = 467). In order to investigate the mechanism of action, a genetic algorithm similarity program (GASP) was employed to construct a pharmacophore model of HBV inhibitors. The binding sites included three hydrophobic points, four HBA points, and one HBD point, and provided possibility for further structural modification.

### HIV Inhibitors

Human immunodeficiency virus (HIV), identified in 1981 as the etiological agent of acquired immunodeficiency syndrome (AIDS), could cause high morbidity and mortality owing to the significant risk of opportunistic infections and malignancy, and become the leading pandemic disease (Ruelas and Greene 2013; Maeda et al., 2019). Because of a large amount of the viruses produced by an infected individual every day, HIV undergoes rapid genetic variation and generates diverse HIV subtypes, which in turn presents moving targets and complicates the development of effective drugs and vaccines (De Cock et al., 2012; Mbonye and Karn 2014).

#### Non-Nucleoside Reverse Transcriptase Inhibitors

Reverse transcriptase (RT), a crucial enzyme regulating the replicative stage of HIV (Kashuba et al., 2012), has been recognized as an attractive target for AIDS treatment. Accordingly, non-nucleoside reverse transcriptase inhibitors (NNRTIs) have been developed with potent anti-HIV-1 activity and favorable pharmacokinetic properties (Daar 2008), and thus gained approval for clinical use (nevirapine, delavirdine, efavirenz, etravirine, and rilpivirine). However, the continued emergence of resistant strains promoted the development of new NNRTIs to inhibit the existing mutants effectively (De Clercq 2001; Daeyaert et al., 2004; Sweeney and Klumpp 2008). Among the structurally diverse NNRTIs, pyridinone scaffolds demonstrated high potency against HIV-1 wild-type and drug-resistant strains.

Benjahad et al. (2004) published a series of articles introducing the discovery of pyridin-2(1*H*)-ones as potent NNRTIs *via* scaffold hopping of Merck pyridinone **l697,661**, a wild-type HIV inhibitor. Considering the pharmacophore of the known ligand (**l697,661**) and its binding mode with the hydrophobic pocket of reverse transcriptase, the benzo[*d*]oxazole moiety was replaced with various heterocyclic aryl rings, and two aromatic rings were linked by the methylene/carbonyl group. In that study, 102 new pyridinone analogs (**70**) were synthesized, and then further evaluated for inhibitory activity against wild-type HIV (LAI cell line) and the K103N, Y181C, and Y188L mutant strains. Among them, thirty-three compounds have been proven to be active at nanomolar range concentrations, and SAR showed that tiny substituent groups at the N-3 motif positively influenced the activity (Figure 8E). In particular, 3′-acrylonitrile-substituted analog **71a** showed the most potent inhibitory activity against four HIV mutant strains, equipotent to that of efavirenz. X-ray crystal structure analysis showed the pyridinone motif engaged in a crucial N-1-H hydrogen-bond with K101, and thus provided a good explanation for the strong binding and good activity against different HIV strains.

On the basis of previous observations, 3-iodo-4-phenoxypyridinone (**71**) was characterized by its potent activity against wild-type HIV-1 reverse transcriptase (Benjahad et al., 2007). The promising biological results inspired further systematic optimization of 3-iodopyridin-2(1*H*)-ones; thus, a 96-member library of 3-iodo-4-phenoxypyridinone (IOPY) and 3-iodo-4-arylthiopyridinones (ISPY) analogs was prepared and evaluated for its *in vitro* activity against wild-type HIV-1 (LAI cell line), K103N, Y181C, and Y188L mutant strains. Due to the geometry/conformation differences between the ISPY and IOPY, even minor structural modifications brought considerable changes in activity. Further biological study was performed to reveal that azole substituents at C5-methyl position exhibited better potency than other functionalized groups. Notably, pyridinone derivatives (**72a**-**c**) showed excellent activity against a panel of strains comparable to the known efavirenz (Figure 8F). Finally, the presented data would serve as a valuable guide to further optimization of the 3-iodopyridinone (IOPY/ISPY) family.

Le Van et al. (2009) reported a series of 4-cycloalkyloxypyridin-2(1*H*)-one derivatives (**73**) with anti-HIV-1 activity. Molecular modeling of derivatives into the allosteric site showed the pyridinone ring was pinned between Leu100 and Val106, allowing the lactam NH to form a hydrogen bond with Lys101, which guaranteed high antiviral activity. In addition, the impact of different ring sizes and substituents of the C4-oxycycloalkyl group in pyridinone was evaluated, and further modulations focused on position 3 of the pyridinone moiety were performed mainly on the basis of molecular modeling studies (Figure 8G). After thorough SAR analysis, compounds **74a** (EC_50_ = 0.001–0.359 μM) and **74b** (EC_50_ = 0.001–0.642 μM) were discovered as most potent molecules against wild-type HIV-1 and several mutant strains, and showed comparable or even superior effect to that of efavirenz (EC_50_ = 0.001–0.525 μM).

Cao et al. (2015) reported a series of novel pyridinone derivatives which were designed based on the known anti-HIV agent **LAM-trans**. Preliminary SAR of the lead compound showed that modifications on positions 3, 4, and 6 of the pyridinone ring were crucial to the antiviral activity (Debnath et al., 2013). Thus, different substituents which were expected to interact with important amino acid residues were introduced at C3 and C4 of the scaffold on account of the molecular docking study. These pyridinone derivatives were synthesized and further evaluated for inhibitory activities by using TZM-bl cell lines. From the results, a partial SAR summarized in Figure 8H confirmed that introduction of the isopropyl moiety on the C-3 would be a great benefit to improve the anti-HIV activity. Notably, **75a** exhibited the best profile against HIV-1 and could be flexibly docked into the binding site of the reverse transcriptase. Because of their excellent antiviral activity, the 6-phenethyl pyridinone family was worthy of further study and exploitation taking the biological and structural data as helpful guidance.

#### Integrase Strand Transfer Inhibitors

HIV-1 integrase (Trono et al., 2010) is an essential enzyme for retrovirus replication and has been established as an attractive antiviral target. Raltegravir, elvitegravir, and dolutegravir are representative marketed HIV-1 integrase strand transfer inhibitors (InSTIs) for their proven efficacy and excellent tolerability (Hare et al., 2010; Smith et al., 2020). In this regard, Seo et al. (2011) designed 1,5-dibenzyl-2-pyridinone (**76**) as an InSTI (IC_50_ = 70 nM), strategically assembled on a pyridinone scaffold. The lead optimization studies were undertaken by introducing various substituents (alkyls, chloro, methoxy, and mixed halo/alkyl) on the phenyl rings, and the related effects on the enzymology involving strand transfer (ST) step were investigated. A focused structure–activity investigation signified the derivatives that possessed fluoro substitution had more compelling ST inhibitory IC_50_ values (<10 nM), which was perhaps induced by hydrophobic and electrostatic interactions (Figure 8I). Notably, compound **76a** emerged from these studies with the most potent inhibition activity and good stability in human liver microsomes. It was also found to possess a remarkable drug interaction profile with cytochrome P450 and human UGTs, as it did not exhibit any inhibition or activation of these isozymes. Because of the poor cellular permeability of **76a**, its isopropyl ester prodrug, compound **76b**, was designed with less polarity and notable therapeutic or selectivity index, and further biological studies are in progress.

## Antibacterial Activity

### Inhibitors of OPRT

**FIGURE 9:**
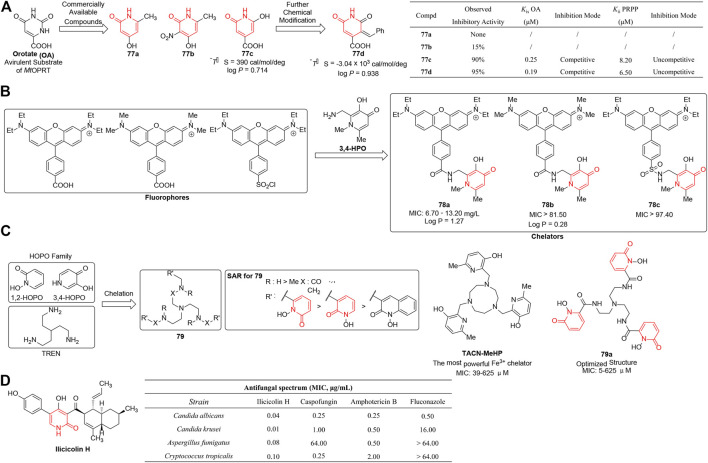
**(A)** 1,2-dihydropyridine-bearing OPRT inhibitors (Breda et al., 2012a). **(B)** 3,4-HPO chelators as antibacterial agents. **(C)** Antibacterial SARs of 1,2-HOPO chelators. **(D)** Antifungal activity of ilicicolin H (Breda et al., 2012b).

Orotate phosphoribosyltransferase (OPRT), an important enzyme catalyzing the synthesis of pyrimidine nucleotides, has been developed as the target for various therapeutic purposes such as tuberculosis (TB), malaria, toxoplasmosis, and tumor (Javaid et al., 1999; Temmink et al., 2007; Abdo et al., 2010; Breda et al., 2012a). Inhibition of mycobacterial cell wall synthesis by the attenuation of *M. tuberculosis* OPRT (*Mt*OPRT) enzyme would be a novel target for TB treatment and prophylaxis. Breda et al. (2012b) reported a series of analogs of orotate (OA) which was a substrate of *Mt*OPRT. Structurally, the commercially available inhibitor **77c** showed submicromolar inhibitory effect on *Mt*OPRT enzyme activity and fit well with the enzyme’s binding pocket according to isothermal titration calorimetry (ITC) data. It was noteworthy that electron withdrawing groups attached on the pyridine-2(1*H*)-one rings contributed to inhibitory effect by increasing the acidity of the hydrogen bound to the nitrogen and thus improving the putative interactions with the active site. However, **77c** was subjected to further optimization due to its unfavorable entropic contribution, detrimental to further pharmaceutical developability. Under the guidance of ITC data, an aromatic ring substituent was added to generate **77d** (Figure 9A), and resulted in entropic optimization which was mainly reflected on a thermodynamic discrimination profile characteristic of high affinity to *Mt*OPRT:PRPP. These derivatives represented lead compounds for further development targeting *Mt*OPRT with increased biological activity and suitable properties for drug development.

### Iron(III) Chelators

As an essential micronutrient needed for microbial metabolism, iron becomes an ideal primary target for metal withdrawal (Breda et al., 2012a). High-affinity iron-selective chelators have a therapeutic benefit in the treatment of infectious diseases by bacterial iron deprivation. Novais et al. (2018) reported a series of fluorescent 3-hydroxy-4-pyridinones (3,4-HPO) chelators as antibacterial agents which were obtained by coupling of the fluorophore to different 3,4-HPO ligands (Figure 9B). Disappointingly, most of the synthesized chelators exhibited inhibitory activity at very high concentrations (MIC: 70 mg/L to 180 mg/L). SAR revealed that the substituents on the nitrogen atom of the fluorophore affected the antibacterial activities a lot, and **78a** was discovered to significantly improve bacteriostasis against Gram-positive strains ranging from 6.7 to 13.2 mg/L. The authors suggested that variable biological activity was probably associated with different lipophilicity which have a powerful influence on the permeability of the cell membrane.

Workman et al. (2020) assumed 1,2-HOPO chelators might have the safety advantages over 3,4-HOPO isomers due to lower pKa of the hydroxyl group and poorer permeability. In 2020, they reported a range of novel 1,2-HOPO chelators which were anchored on a tris(2-aminoethyl)amine (TREN) core (**79**) and the effect of different chelators on microbial growth. The results revealed that most compounds existed as powerful metal chelators and stunted the growth of bacteria by Fe3+ starvation. Among all derivatives, compound **79a** was the most potent agent (MIC = 5–625 μM), almost comparable to that of TACN-MeHP (MIC = 39–625 μM) which was known as the most powerful Fe^3+^ chelator. As the SAR shown in Figure 9C, the nature and position of the linkers between 1,2-HOPO and TREN core strongly influenced the growth of microorganisms by iron(III) starvation, and both amide and amine linkages ensured optimal activity.

### Antifungal Activity

Singh et al. (2012) reported the discovery of ilicicolin H as a novel antifungal agent by screening natural product extracts isolated from *Gliocladium roseum*. The biological assay revealed that the compound exhibited potent inhibition against a wide range of fungi which was even better than those of the reference comparators including caspofungin, amphotercin B, and fluconazole (Figure 9D). In addition, ilicicolin H took effect by depressing mitochondrial cytochrome bc1 reductase (IC_50_ = 2–3 ng/ml) which represented an unique mode of action, and the *in vivo* efficacy of the inhibitor was modest due to the limitation by high plasma protein binding. Further structural modification and biological data suggested that hydroxyl-pyridinone was very crucial for antifungal activity.

## Central Nervous System-Related Activity

### AD Therapeutic Agents

Alzheimer’s disease (AD) is a neurodegenerative disorder (Hodson 2018) which would lead to typical symptoms like memory loss and cognitive impairment. However, the multifactorial and complex etiologies including amyloid-β (Aβ) deposits, tau protein hyperphosphorylation, metal ion dyshomeostasis, oxidative stress, and neurotransmitter system dysfunction have limited the therapeutic effects (León et al., 2013). On account of this, the multi-target-directed ligand (MTDL) strategy has been developed and provides promising therapeutic effects.

**FIGURE 10:**
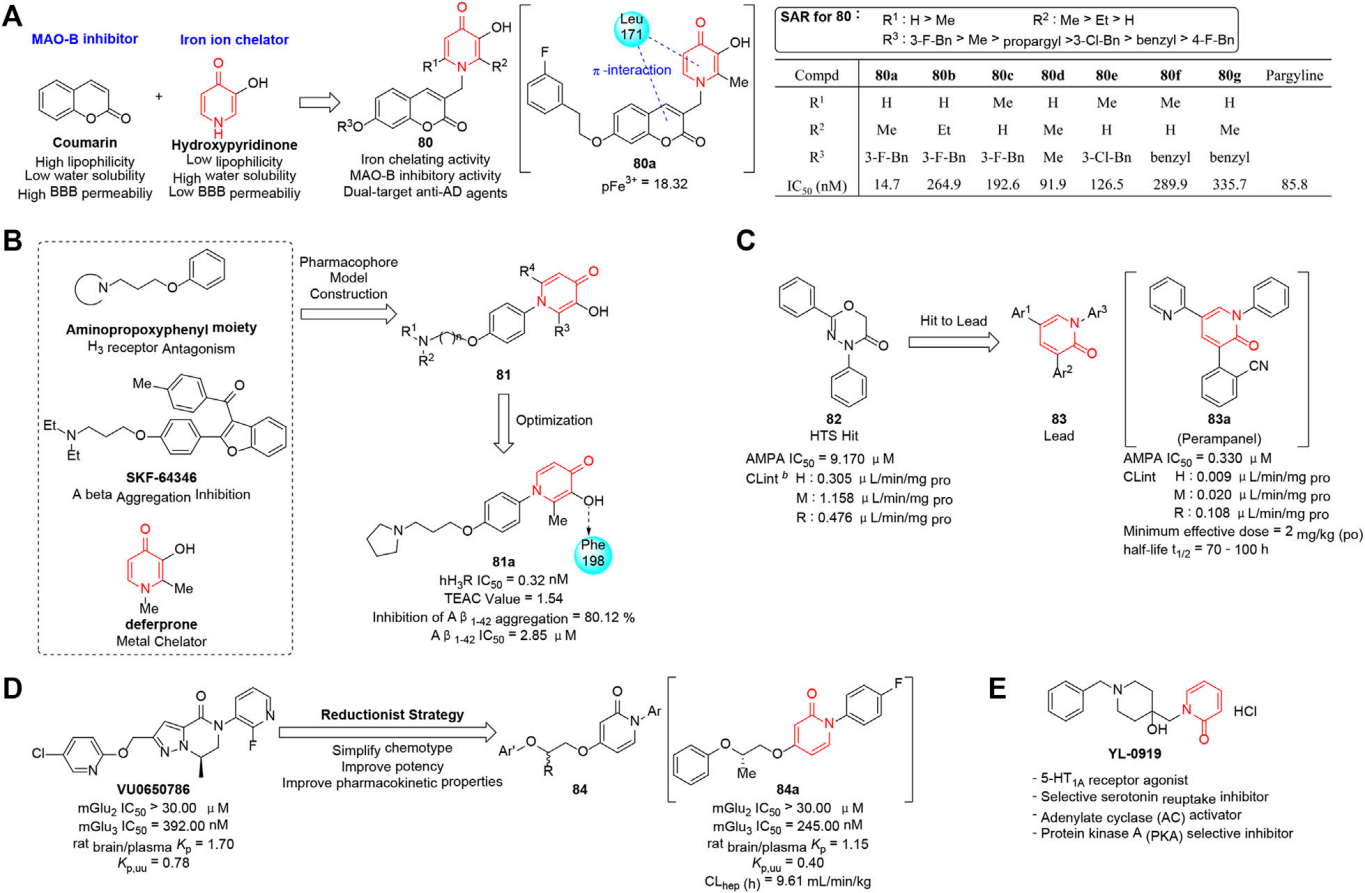
**(A)** Strategy and SAR of dual-target anti-AD agents (Zhang et al., 2019). **(B)** Discovery of pyridinone-based anti-AD agents. **(C)** Optimization of pyridinone-based AMPA-type glutamate receptor antagonists. **(D)** *N*-aryl phenoxyethoxypyridinones as highly selective mGlu_3_ NAMs. **(E)** Structure of YL-0919.

Zhang et al. (2019) designed and synthesized a series of (3-hydroxypyridin-4-one)-coumarin hybrids (**80**) targeting iron ion and monoamine oxidase B (MAO-B) cooperatively for the treatment of AD. The biological evaluation showed most compounds exhibited good iron-chelating effects (pFe^3+^ ≈ 18) as well as moderate to excellent anti-MAO-B activities. As summarized in Figure 10A, the SAR was revealed and potency was enhanced by modification at C-7 of the coumarin ring. In particular, compound **80a** possessed the most promising MAO-B inhibitory activity (IC_50_ = 14.7 nM) and good U251 cell protective effect which contributed to the amelioration of cognitive dysfunction in a Morris water maze test model. Moreover, molecular docking analysis elucidated **80a** could bind to both the entrance and the substrate cavity of MAO-B, indicating that the development of agents with multi-target-directed targets was a useful strategy for treating AD.

As quadruple functional agents for the therapy of AD, a series of novel 1-phenyl-3-hydroxy-4-pyridinone derivatives (**81**) targeting H_3_ receptor, Aβ peptide, metal ion, and radical simultaneously were reported by Hu’s group (2016). As shown in Figure 10B, compound **81** were designed by combining deferiprone (DFP) and aminopropoxyphenyl into one molecule through rational pharmacophore model construction. Biological evaluation indicated that **81a** exhibited the most potent H_3_ receptor antagonistic activity (IC_50_ = 0.32 nM), efficient radical scavenging activity (Trolox-equivalent antioxidant capacity value = 1.54), excellent Aβ self-aggregation inhibitory activity (IC_50_ = 2.85 μM), and good chelating properties with copper and iron. The four functions mentioned earlier promoted **81a** to be a potential candidate for the AD treatment. Moreover, an *in vivo* study demonstrated **81a** possessed appropriate pharmacokinetic profiles and the proper ability to permeate the blood–brain barrier (BBB) efficiently. This work provided an attractive strategy to develop potential multifunctional agents with various mechanisms.

### Glutamate Receptor Antagonists

Glutamate (Niswender and Conn 2010), the primary excitatory neurotransmitter in the central nervous system, is associated with a series of neurological diseases including epilepsy which is triggered by high concentration of glutamate in the brain. Therefore, glutamate receptor antagonists are explored to block the corresponding gating. Hibi et al. (2012) discovered **82** as an initial hit by applying the high-throughput screening strategy, and then chemical modification was carried out to improve the stability. Thus, **82** was transformed to 1,3,5-triaryl-1*H*-pyridin-2-one derivatives (**83**) that could non-competitively inhibit the activity of α-amino-3-hydroxy-5-methyl-4-isoxazolepropionic acid receptor (AMPAR) which is known as one type of ionotropic glutamate receptors (iGluRs). As shown in Figure 10C, an activity-guided SAR optimization study was performed by manipulating individual aryl groups at 1,3,5-positions of pyridinone ring, and 2-(2-oxo-1-phenyl-5-pyridin-2-yl-1,2-dihydropyridin-3-yl)benzonitrile (**83a**, perampanel) was revealed be an extremely potent non-competitive AMPA receptor antagonist (IC_50_ = 60 nM). In addition, **83a** possessed powerful *in vivo* activity in preclinical AMPA-induced seizure models (ED_50_ = 0.47–1.60 mg/kg, minimum effective dose of 2 mg/kg po) and favorable pharmacokinetic characteristics in early clinical studies (CL_int_ = 0.009 μL min^−1^ mg^−1^). It is gratifying that the compound has been approved by the FDA for treating patients with epilepsy in 2012.

G-protein-coupled metabotropic glutamate receptors (mGluRs) have emerged as validated therapeutic targets with potential for modulating prefrontal cortex (PFC) glutamate transmission. Engers et al. (2017) reported a series of pyrimidinone and pyridinone derivatives (**84**) as mGlu_3_ negative allosteric modulators (NAMs). The basic design of the scaffold was originated from **VU0650786** which emerged as a valuable mGlu_3_ NAM *in vivo* probe with a complex molecular structure, cumbersome multistep synthetic procedure, and unsatisfactory physicochemical properties. To optimize the scaffold, a more flexible linker and an *N*-arylpyrimidine or *N*-arylpyridine head-piece were introduced through a reductionist strategy, and 12 analogs were synthesized and tested for their potency (Figure 10D). Finally, **84a** was identified as the most effective mGlu_3_ NAM (IC_50_ = 245 nM) with excellent CNS penetration (rat brain/plasma K_p_ = 1.2) and displayed highly selective *in vivo* antidepressant activity (inactive at mGlu_1,4,6,7,8_) in a mouse tail suspension test (MED = 3 mg/kg).

### A Dual 5-HT_1A_ Receptor Agonist and Serotonin Uptake Inhibitor

5-HT_1A_ receptor (Brummelte et al., 2017; Cortes-Altamirano et al., 2018; Żmudzka et al., 2018), an inhibitory G-protein-coupled receptor in the CNS, is implicated in a number of behavioral traits associated with anxiety and depression-like disorders. Improvement of 5-HT levels in the synaptic gap by regulating activity of 5-HT_1A_ receptor and suppressing 5-HT reuptake provides promising therapeutic targets for the treatment of such mood disorders (Kaufman et al., 2016; Brummelte et al., 2017). Li’s group (2014) reported a novel antidepressant candidate YL-0919 with both 5-HT reuptake inhibitory effect and 5-HT_1A_ receptor emotional activity (Figure 10E). The antidepressant effect and mechanism were evaluated by the animal model test and cAMP assays, and the results demonstrated that YL-0919 could remarkably reverse the depressive-like behaviors in a dose-dependent manner through the activation of the AC-cAMP-PKA signal-transduction passway in the frontal cortex. Like vilazodone, an FDA-approved drug sharing the same mechanisms as YL-0919, drugs with dual pharmacological effects on both serotonin transporter and 5-HT_1A_ receptor have become the principal focus of research and development of novel antidepressant.

## Anti-Inflammatory and Analgesic Activity

### p38α Kinase Inhibitors

**FIGURE 11:**
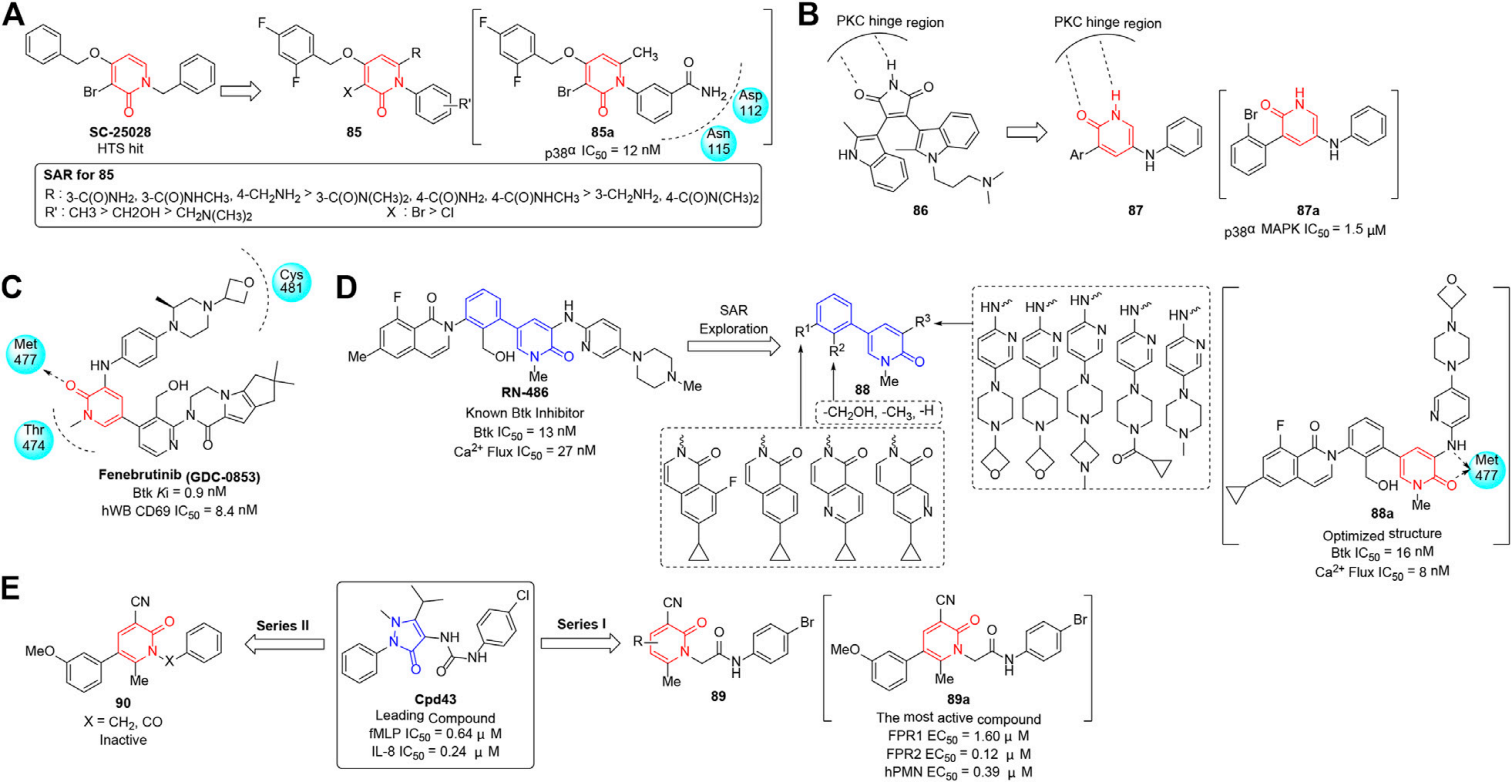
**(A)** N-aryl pyridinones with selective p38α inhibitory activity. **(B)** Pyridin-2(1*H*)-one analogs with p38α MAPK inhibitory activity. **(C)** Structure of fenebrutinib and its binding mode to Btk (PDB code 5VFI). **(D)** Optimization and PK properties study for pyridinone-based Btk inhibitors. **(E)** Two series of pyridinone-based FPR agonists.

Tumor necrosis factor-α (TNF-α) is an important pro-inflammatory cytokine (Dingar et al., 2010), and its elevated level correlates with a broad range of inflammatory diseases such as rheumatoid arthritis (RA), psoriasis, and Crohn’s disease. Among a number of stress-activated protein kinases associated with TNF-α, p38α is validated as a potential therapeutic target (Wolańska et al., 2010; Ma et al., 2016; Slobodnyuk et al., 2019) that could act effectively on the cytokine expression and subsequently alleviate the inflammation. Selness et al. (2011) developed *N*-aryl pyridinones as selective p38α inhibitor chemotypes, which were identified from the previous discovery of the initial lead SC-25028 screened from a high-throughput full file screen. Structural modification was based on the key binding contacts between SC-25028 and p38α, and a series of *N*-aryl derivatives (**85**) were synthesized and evaluated for inhibitory activity, stability, and pharmacokinetic profiles (Figure 11A). The cascade assay findings proved that most analogs demonstrated robust inhibitory activity against p38α at nanomolar concentrations. In particular, compound **85a** showed remarkable cellular activity (IC_50_ = 12 nM) with satisfactory metabolic stability, and exhibited potency in both acute and chronic models of inflammation.

As an important subtribe of mitogen-activated protein kinases (MAPKs), p38α MAPK has been proven to contribute to pain hypersensitivity (Jensen and Finnerup 2014) associated with tactile inputs in the spinal dorsal horn (SDH) or medullary dorsal horn (MDH). Therefore, inhibitors of p38α MAPK are proposed to alleviate chronic pain syndromes including neuropathic and inflammatory pain (Liu et al., 2017; He et al., 2018; Canovas and Nebreda 2021). Previously, (2-methyl-indol)-BIM-1 (**86**) was recognized as a potent protein kinase C (PKC) inhibitor by Grant’s group. Visseq et al. (2020) reported a novel series of 3,5-disubstituted pyridin-2(1*H*)-ones (**87**), which were designed based on the structure of **86** whose central core formed H-bond interactions with the hinge region of PKC (Figure 11B). Antiallodynic activity was evaluated for the synthesized compounds using Complete Freund’s Adjuvant (CFA) models, and most of the derivatives prevented the development of mechanical allodynia. SAR showed that the 3-position of the pyridinone moiety tolerated bulky groups, while substitution at the 2-position by a bromine atom or an ethoxycarbonyl group was favorable. Finally, compound **87a** was suggested as a candidate p38α MAPK inhibitor (IC_50_ = 1.5 μM) in terms of the rapid response ability to reverse facial neuropathic allodynia in rats.

### Bruton’s Tyrosine Kinase Inhibitors

Bruton’s tyrosine kinase (Btk), a Tec family tyrosine kinase, is a critical effector molecule in hematopoietic cells and plays multiple roles in B-cell development (Crawford et al., 2018). Overexpression of Btk may lead to B-cell lymphoproliferative disorders (Pal Singh et al., 2018), such as hematologic malignancies and autoimmune diseases including rheumatoid arthritis (RA), systemic lupus erythematosus (SLE), and multiple sclerosis (MS). Therefore, the kinase has been identified as an emerging therapeutic target for the treatment of cancer and immune system dysfunction (Pal Singh et al., 2018). Decades of research have advanced several Btk inhibitors for clinical evaluation; among them, fenebrutinib (GDC-0853), structurally based on an amino pyridone hinge scaffold (Johnson et al., 2016), is a notable one that binds to the ATP-binding site of Btk without interacting with Cys481 (Figure 11C). In addition, fenebrutinib (Young et al., 2015) possesses high selectivity, potential efficacy, and favorable pharmacokinetic (PK) and pharmacodynamic (PD) profiles, and is currently under phase 2 investigation for patients with RA (NCT02833350).

**RN486**, a novel reversible Btk inhibitor discovered by Roche, exhibited perfect activity and high selectivity, and displayed satisfactory inhibition of arthritis in animal models. As illustrated in Figure 11D, Zhao et al. (2015) took advantage of 5-phenylpyridin-2(1*H*)-one skeleton which was derived from RN486 as a privileged scaffold, and modified it on R^1^, R^2^, and R^3^, thus investigating a novel series of reversible Btk inhibitors (**88**) in 2015. The bioactivity was evaluated by measuring the *in vitro* enzymatic and cellular (Ramos cell) inhibition, and the SAR showed that the hydroxyl group on R^2^ was crucial for good potency. Moreover, derivative **88a** exhibited excellent PK properties and perfect *in vivo* efficacy of inhibiting arthritis in the collagen-induced arthritis (CIA) model.

### Formyl Peptide Receptor Agonists

Formyl peptide receptor (FPR), a target for specific pro-resolving mediators (SPMs), acts in dual effects by performing pro-resolving and anti-inflammatory activities, and contributes to the resolution of inflammation (Stama et al., 2017). Previously, **Cpd43** (Odobasic et al., 2018), a mixed FPR1/FPR2 agonist, was recognized as an effective therapeutic agent for rheumatoid arthritis developed by Amgen. Crocetti et al. (2020) expanded the imidazolone of **Cpd43** to pyridinone scaffold and reported a series of derivatives (**89**) containing a 4-bromophenylacetamide fragment which was proven to be crucial for the potency. They evaluated the FPR emotional activity of the synthesized compounds by measuring intracellular Ca^2+^ flux in transfected cells and revealed that the majority of analogs exhibited effective mixed FPR agonist activity in the submicromolar/micromolar range. A partial SAR was revealed in Series I (**89**), where the position and types of substituents at pyridinone rings had a great influence on the properties of FPR excitatory, while the replacement of 4-bromophenylacetamide chain in Series II (**90**) led to completely inactive compounds (Figure 11E). In particular, **89a** was validated as the most active structure, with EC_50_ value of 1.60 μM for FPR1 and 0.12 μM for FPR2. It could also directly induce neutrophil chemotaxis and inhibit WKYMVm-induced chemotaxis *via* desensitization. *In vivo* evaluation of **89a** exhibited similar efficacy with ibuprofen and a longer lasting effect in a rat model of rheumatoid arthritis.

## Anticoagulants

**FIGURE 12:**
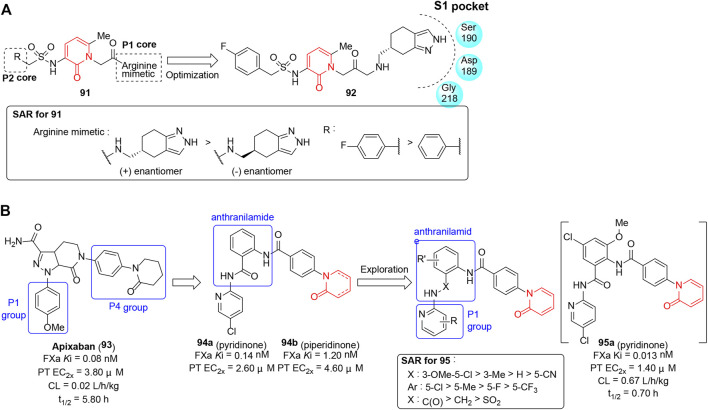
**(A)** SAR of pyridinone as thrombin inhibitors. **(B)** Pyridinones with FXa inhibitory activity.

Dysregulation of blood coagulation results in various thromboembolic disorders with inappropriate formation of fibrin, subsequently causing tissue ischemia (Chapin and Hajjar 2015). Serine proteases including factor Xa (FXa), factor XIa (FXIa), factor VII (FVII)/tissue factor (TF), and thrombin play a pivotal role in the amplification of thrombin production and are considered potential targets for anticoagulant therapy (Melnikova 2009; Bagoly et al., 2012). The pyridinone template has been proven to be an appropriate peptidomimetic template in the design of thrombin inhibitors and tissue factor/factor VIIa inhibitors due to its ability to mimic the hydrogen bond array of the backbone of peptide inhibitors and provides a good fit of the inhibitor in the enzyme active site. With the intention to improve the receptor binding affinity and selectivity, Kranjc et al. (2005) reported a series of novel thrombin inhibitors (**91**) containing a pyridinone core and (±)-4,5,6,7-tetrahydro-2*H*-indazol-5-ylmethanamine as P1 arginine side-chain mimetics. Each compound’s *in vitro* inhibitory potency was measured by amidolytic enzyme assay, and a partial SAR shown in Figure 12A indicated that the binding affinity was closely related to the absolute configuration. Finally, (+)-enantiomer **92** (thrombin *K*i = 0.047 μM) was optimal and the X-ray co-crystal structure of **92** bound to human thrombin revealed its bicyclic ring fit well into the S1 pocket, while the central aromatic P2 core and the sulfonyl group exhibited only weak influence on binding of the inhibitor to the thrombin active site.

Corte et al. (2008) reported anthranilamide-pyridinone hybrids (**95**) as selective FXa inhibitors. Initially, hits (**94**) were discovered by introducing the anthranilamide scaffold into the clinical drug apixaban and incorporating the phenyl piperidinone or pyridinone in P4 group (Figure 12B). SAR revealed incorporation of the unsaturated version, the phenyl pyridinone which formed an edge to face interaction with W215, and was appropriately sandwiched between Y99 and F174; it promoted **94a** as a potent fXa inhibitor with similar *in vitro* activity to apixaban. Further structural optimization which focused on the exploration of P1 group and anthranilamide scaffold generated **95a** as a novel fXa inhibitor that showed the best profiles in terms of potency, selectivity, and oral bioavailability. However, **95a** exhibited a shorter *in vivo* half-life than apixaban in a dog pharmacokinetics study due to its higher clearance.

## Antimalarial Activity

**FIGURE 13:**
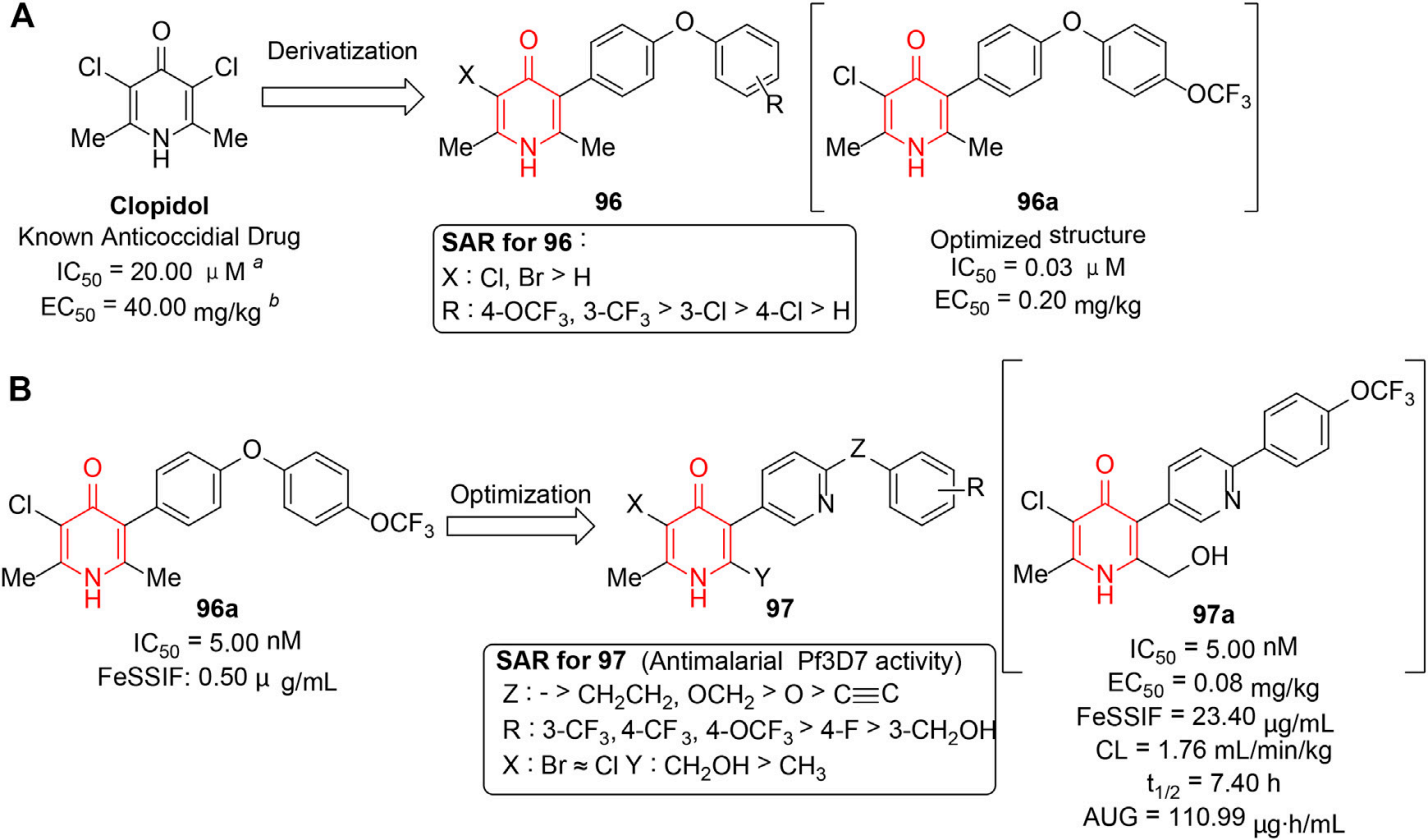
**(A)** Design of 4(1*H*)-pyridinone hybrids based on a known active ligand. **(B)** Antimalarial SARs of 5-pyridinyl-4(1*H*)-pyridinone derivatives.

Plasmodium malaria (Lubell et al., 2014) is a prevalent and devastating parasitic disease due to its high virulence and drug resistance. As a consequence, it is an urgent requirement for the development of new antimalarial drugs with novel chemotypes. Cytochrome *bc*1 (Barton et al., 2010; Fisher et al., 2020) has played a prominent part in mitochondrial respiratory chain of *Plasmodium falciparum*. Hence, specific impairment of mitochondrion by binding to the site of cytochrome *bc*1 could provide an attractive option for antimalarial drugs. In this regard, Leon et al. (2008) reported the synthesis and biological evaluation of a series of 4(1*H*)-pyridinone hybrids (**96**) which were derived from anti-coccidial drug clopidol containing the same core developed by GSK. Structurally, the desired inhibitors were afforded *via* incorporation of lipophilic side chains into clopidol, yielding a number of derivatives with significant improvement in potency (Figure 13A). Candidate compound **96a** exhibited 500-fold increased *in vitro* activity compared to clopidol and selectively inhibited respiration by acting upon the cyt*bc1* complex. Although the poor solubility and oral bioavailability of derivatives did not meet the requirements for therapeutic use, the research has encouraged the further exploration of this series as potential antimalarials.

In 2018, the same group (Bueno et al., 2018) performed the follow-up exploration starting from the previously developed antagonists **96**. A series of novel “hybrid” polar 4(1*H*)-pyridinone derivatives (**97**) were obtained by introducing pyridine rings into lipophilic side chains or attaching polar moieties such as hydroxymethyl group to the 4(1*H*)-pyridinone cores. The results revealed that most new compounds displayed improved pharmacokinetic profiles including physicochemical properties and oral bioavailabilities, and maintained excellent potency as **96a** *in vitro* and *in vivo*. The selected results for partial SAR are summarized in Figure 13B, which showed that 1) rigid linkers between two aromatic rings exhibited better activity than the flexible ones; 2) as for phenyl substituents, electron-withdrawing groups such as CF_3_ or OCF_3_ enhanced activity; and 3) the effect of the type of halogens at C3-pyridinone could be negligible. Eventually, **97a** was suggested as the most efficacious antimalarial, with a long half-life and a high AUC. The novel compounds widened the scope of the antimalarial 4(1*H*)-pyridinones available, thus opening new ways for the chemical exploration of this exciting family of antimalarials.

## Cardiotonic Activity

**FIGURE 14:**
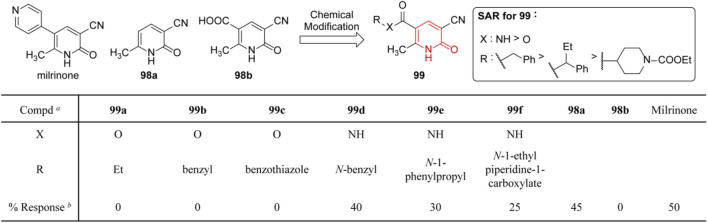
Cardiotonic SARs of 3-cyano-6-methyl-2(1*H*)-pyridinone (Pietrangelo et al., 2010). ^*a*^ Percentage of differentiated H9C2 that prolonged the KCl-dependent [Ca^2+^]_i_ transient in the presence of new compounds and milrinone. ^*b*^ Concentration of 10 μM.

Heart failure (Bueno et al., 2018) is considered to be the major cause of death in patients with cardiac disease. Among therapeutic targets strengthening myocardial contraction, phosphodiesterase-3 (PDE3) is a validated one that could increase calcium influx in cardiac myocytes and trigger positive inotropic effects, and agents such as milrinone and amrinone have been approved for clinical use (Thompson et al., 2007). Pietrangelo et al. (2010) reported a series of 2(1*H*)-pyridinone (**99**) as analogs of milrinone, in which the pyridyl moiety was replaced with an ester or amide group based on the docking study between PDE3 crystal structure and published antagonisms (**98a** and **98b**). In order to evaluate the efficiency of **99**, the degree of calcium channel activation during the plasma membrane depolarization in H9C2 cardiomyocytes was evaluated through the calcium imaging assay, and partial SARs revealed amide derivatives exhibited more potent activity than esters (Figure 14). The authors attributed this to the better resistance of amides under hydrolysis conditions, while the esters were probably cleaved to afford the inactive acid. As a result, among milrinone analogs, only amide derivatives could support intracellular [Ca^2+^] influx following chemical depolarization.

## EP_3_ Receptor Antagonists

**FIGURE 15:**
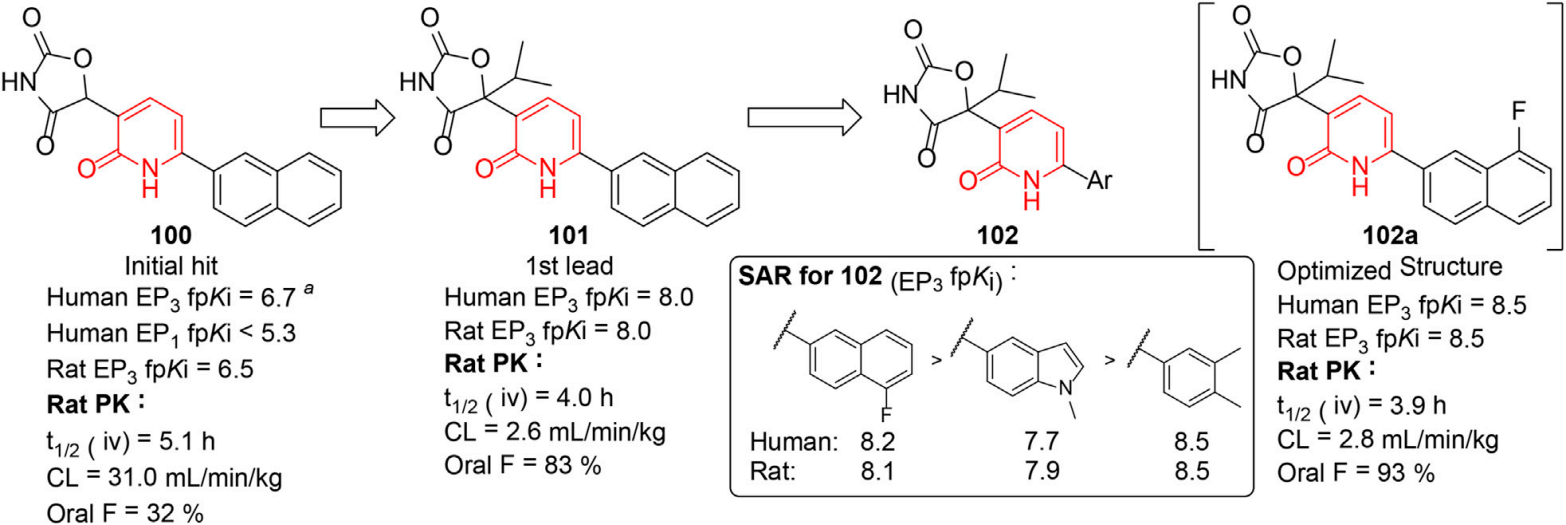
EP_3_ inhibitory activities of 3-oxazolidinedione-6-aryl-pyridinones (Jin et al., 2010). ^*a*^ Value of functional p*K*i(fp*K*i) was obtained from a EP_3_ fluorometric imaging plate reader (FLIPR) assay.

Overactive bladder (OAB) is a symptom syndrome of urinary urgency which is generally accompanied by frequency and nocturia (Abrams et al., 2003). Prostaglandin EP_3_ receptor (Chen and Kuo 2019) could regulate the excitability of bladder smooth muscle and is considered a potential target for OAB. Jin et al. (2010) reported a series of 3-oxazolidinedione-6-aryl-pyridinones (**102**) as selective EP_3_ receptor antagonists, generated by the optimization of **100** and **101** which were identified through the high-throughput screening (HTS) method. A thorough investigation on **101** indicated the lead compound exhibited excellent pharmacokinetic profiles and robust potency in several overactive bladder models (Figure 15). But the unsubstituted naphthyl moiety of **101** could be oxidized to generate reactive metabolites in glutathione trapping studies. To address such potential bioactivation liability, a substituted aryl group was introduced to the lead compound, resulting in the discovery of compound **102a** with improved stability and excellent efficacy. In addition, these highly potent, selective, and orally bioavailable compounds were beneficial tool compounds for developing and validating potential therapeutic benefits arising from selective EP_3_ inhibition.

## Conclusion and Future Perspectives

In summary, we described significant roles that pyridinone scaffolds played in medicinal chemistry owing to their wide range of pharmacological properties. Most of the drug discovery projects presented in this work benefited from the chemical diversifiability and convenient synthesis method of target groups. Structurally, N1, C3, C5, and C6 of the pyridinone scaffold could be easily functionalized *via* C-H or N-H activation and produces corresponding pyridinone-containing compounds. These compounds exhibit various biological activities in low concentrations (often submicromolar), such as cytotoxic, antibacterial, anti-inflammatory, antiviral, antimalarial, anticoagulant, and psychotropic activities, and some have known SAR and mechanisms of action (their effect on certain enzymes, receptors, *etc*.), which makes them potentially useful in pharmaceutic research. In addition, the pyridinone moiety serves as key binders between two pharmacophores, and improves pharmacokinetics by altering the solubility and selectively interacting with the enzyme’s binding site. The ongoing studies covering various fields of medicinal chemistry have also revealed several small pyridinone analogs with notable biological activity, and some of them are under clinical trials and possess brilliant application prospects. These advancements contribute to an in-depth understanding of the potential of this biologically enriched scaffold and pave the way to apply the prospective novel pyridinone-based derivatives for further rational development in drug discovery.

## References

[B1] Abaszadeh M. Sheibani H. Saidi K. (2009). The Reaction of (Chlorocarbonyl)phenyl Ketene with Enaminones: A Novel Synthesis of Some 5-Acyl-4-Hydroxy-2-(1h)-Pyridinones and 7-Hydroxy-5-Oxo-1,4-Diazepin Derivative. J. Heterocyclic Chem. 46, 96–99. 10.1002/jhet.14

[B2] Abdo M. Zhang Y. Schramm V. L. Knapp S. (2010). Electrophilic Aromatic Selenylation: New OPRT Inhibitors. Org. Lett. 12, 2982–2985. 10.1021/ol1010032 20521773 PMC2906230

[B3] Abou-Elkhair R. A. I. Moustafa A. H. Haikal A. Z. Ibraheem A. M. (2014). Synthesis and Biological Evaluation of 2-oxonicotinonitriles and 2-oxonicotinonitrile Based Nucleoside Analogues. Eur. J. Med. Chem. 74, 388–397. 10.1016/j.ejmech.2013.12.055 24486419 PMC7115408

[B4] Abrams P. Cardozo L. Fall M. Griffiths D. Rosier P. Ulmsten U. (2003). The Standardisation of Terminology in Lower Urinary Tract Function: Report from the Standardisation Sub-committee of the International Continence Society. Urology 61, 37–49. 10.1016/s0090-4295(02)02243-4 12559262

[B5] Agostinis P. Berg K. Cengel K. A. Foster T. H. Girotti A. W. Gollnick S. O. (2011). Photodynamic Therapy of Cancer: an Update. CA: A Cancer J. Clinicians 61, 250–281. 10.3322/caac.20114 PMC3209659 21617154

[B6] Alafeefy A. M. Alqasoumi S. I. Ashour A. E. Masand V. Al-Jaber N. A. Ben Hadda T. (2012). Quinazoline-tyrphostin as a New Class of Antitumor Agents, Molecular Properties Prediction, Synthesis and Biological Testing. Eur. J. Med. Chem. 53, 133–140. 10.1016/j.ejmech.2012.03.044 22520152

[B7] Ang Y.-S. Tsai S.-Y. Lee D.-F. Monk J. Su J. Ratnakumar K. (2011). Wdr5 Mediates Self-Renewal and Reprogramming via the Embryonic Stem Cell Core Transcriptional Network. Cell 145, 183–197. 10.1016/j.cell.2011.03.003 21477851 PMC3097468

[B8] Atkins J. H. Gershell L. J. (2002). Selective Anticancer Drugs. Nat. Rev. Drug Discov. 1, 491–492. 10.1038/nrd842 12120255

[B9] Bagoly Z. Koncz Z. Hársfalvi J. Muszbek L. (2012). Factor XIII, Clot Structure, Thrombosis. Thromb. Res. 129, 382–387. 10.1016/j.thromres.2011.11.040 22197181

[B10] Bai F. Hu D. Liu Y. Wei L. (2018). One-Pot and Multicomponent Synthesis of N-Substituted-4-Hydroxyl-2-Pyridones. Chin. J. Org. Chem. 38, 2054–2059. 10.6023/cjoc201801015

[B11] Bailey J. K. Fields A. T. Cheng K. Lee A. Wagenaar E. Lagrois R. (2015). WD Repeat-Containing Protein 5 (WDR5) Localizes to the Midbody and Regulates Abscission. J. Biol. Chem. 290, 8987–9001. 10.1074/jbc.M114.623611 25666610 PMC4423688

[B12] Barton V. Fisher N. Biagini G. A. Ward S. A. O’Neill P. M. (2010). Inhibiting Plasmodium Cytochrome Bc1: a Complex Issue. Curr. Opin. Chem. Biol. 14, 440–446. 10.1016/j.cbpa.2010.05.005 20570550

[B13] Battah S. Hider R. C. MacRobert A. J. Dobbin P. S. Zhou T. (2017). Hydroxypyridinone and 5-Aminolaevulinic Acid Conjugates for Photodynamic Therapy. J. Med. Chem. 60, 3498–3510. 10.1021/acs.jmedchem.7b00346 28363026

[B14] Benjahad A. Courté K. Guillemont J. Mabire D. Coupa S. Poncelet A. (2004). 4-benzyl- and 4-Benzoyl-3-Dimethylaminopyridin-2(1h)-Ones, a New Family of Potent Anti-HIV Agents: Optimization and *In Vitro* Evaluation against Clinically Important HIV Mutant Strains. J. Med. Chem. 47, 5501–5514. 10.1021/jm0407658 15481987

[B15] Benjahad A. Oumouch S. Guillemont J. Pasquier E. Mabire D. Andries K. (2007). Structure-activity Relationship in the 3-Iodo-4-Phenoxypyridinone (IOPY) Series: The Nature of the C-3 Substituent on Anti-HIV Activity. Bioorg. Med. Chem. Lett. 17, 712–716. 10.1016/j.bmcl.2006.10.082 17157017

[B16] Bergeron R. J. McManis J. S. Franklin A. M. Yao H. Weimar W. R. (2003). Polyamine−Iron Chelator Conjugate. J. Med. Chem. 46, 5478–5483. 10.1021/jm0302694 14640556

[B17] Bhattacharya D. Thio C. L. (2010). Review of Hepatitis B Therapeutics. Clin. Infect. Dis. 51, 1201–1208. 10.1086/656624 20954965 PMC2977969

[B18] Bobrowski T. Melo-Filho C. C. Korn D. Alves V. M. Popov K. I. Auerbach S. (2020). Learning from History: Do Not Flatten the Curve of Antiviral Research!. Drug Discov. Today 25, 1604–1613. 10.1016/j.drudis.2020.07.008 32679173 PMC7361119

[B19] Borger D. R. Tanabe K. K. Fan K. C. Lopez H. U. Fantin V. R. Straley K. S. (2012). Frequent Mutation of Isocitrate Dehydrogenase (IDH)1 and IDH2 in Cholangiocarcinoma Identified through Broad-Based Tumor Genotyping. Oncologist 17, 72–79. 10.1634/theoncologist.2011-0386 22180306 PMC3267826

[B20] Brackett C. M. Gollnick S. O. (2011). Photodynamic Therapy Enhancement of Anti-tumor Immunity. Photochem. Photobiol. Sci. 10, 649–652. 10.1039/c0pp00354a 21253659 PMC3197776

[B21] Breda A. Machado P. Rosado L. A. Souto A. A. Santos D. S. Basso L. A. (2012). Pyrimidin-2(1H)-ones Based Inhibitors of *Mycobacterium tuberculosis* Orotate Phosphoribosyltransferase. Eur. J. Med. Chem. 54, 113–122. 10.1016/j.ejmech.2012.04.031 22608674

[B22] Breda A. Rosado L. A. Lorenzini D. M. Basso L. A. Santos D. S. (2012). Molecular, Kinetic and Thermodynamic Characterization of Mycobacterium Tuberculosisorotate Phosphoribosyltransferase. Mol. Biosyst. 8, 572–586. 10.1039/c1mb05402c 22075667

[B23] Brondel N. Renoux B. Gesson J.-P. (2006). New Strategy for the Synthesis of Phosphatase Inhibitors TMC-69-6H and Analogs. Tetrahedron Lett. 47, 9305–9308. 10.1016/j.tetlet.2006.10.102

[B24] Brummelte S. Mc Glanaghy E. Bonnin A. Oberlander T. F. (2017). Developmental Changes in Serotonin Signaling: Implications for Early Brain Function, Behavior and Adaptation. Neuroscience 342, 212–231. 10.1016/j.neuroscience.2016.02.037 26905950 PMC5310545

[B25] Bryan M. C. Rajapaksa N. S. (2018). Kinase Inhibitors for the Treatment of Immunological Disorders: Recent Advances. J. Med. Chem. 61, 9030–9058. 10.1021/acs.jmedchem.8b00667 29870256

[B26] Bueno J. M. Calderon F. Chicharro J. De la Rosa J. C. Díaz B. Fernández J. (2018). Synthesis and Structure-Activity Relationships of the Novel Antimalarials 5-Pyridinyl-4(1h)-Pyridones. J. Med. Chem. 61, 3422–3435. 10.1021/acs.jmedchem.7b01256 29589932

[B27] Canovas B. Nebreda A. R. (2021). Diversity and Versatility of P38 Kinase Signalling in Health and Disease. Nat. Rev. Mol. Cel Biol 22, 346–366. 10.1038/s41580-020-00322-w PMC7838852 33504982

[B28] Cao Y. Zhang Y. Wu S. Yang Q. Sun X. Zhao J. (2015). Synthesis and Biological Evaluation of Pyridinone Analogues as Novel Potent HIV-1 NNRTIs. Bioorg. Med. Chem. 23, 149–159. 10.1016/j.bmc.2014.11.012 25468035

[B29] Caravella J. A. Lin J. Diebold R. B. Campbell A.-M. Ericsson A. Gustafson G. (2020). Structure-Based Design and Identification of FT-2102 (Olutasidenib), a Potent Mutant-Selective IDH1 Inhibitor. J. Med. Chem. 63, 1612–1623. 10.1021/acs.jmedchem.9b01423 31971798

[B30] Chapin J. C. Hajjar K. A. (2015). Fibrinolysis and the Control of Blood Coagulation. Blood Rev. 29, 17–24. 10.1016/j.blre.2014.09.003 25294122 PMC4314363

[B31] Chen L. C. Kuo H. C. (2019). Pathophysiology of Refractory Overactive Bladder. Lower Urinary Tract Symptoms 11, 177–181. 10.1111/luts.12262 30900373

[B32] Chen W. Chen X. Li D. Zhou J. Jiang Z. You Q. (2021). Discovery of DDO-2213 as a Potent and Orally Bioavailable Inhibitor of the WDR5-Mixed Lineage Leukemia 1 Protein-Protein Interaction for the Treatment of MLL Fusion Leukemia. J. Med. Chem. 64, 8221–8245. 10.1021/acs.jmedchem.1c00091 34105966

[B33] Cho M. E. Kopp J. B. (2010). Pirfenidone: an Anti-fibrotic Therapy for Progressive Kidney Disease. Expert Opin. Investig. Drugs 19, 275–283. 10.1517/13543780903501539 PMC3058482 20050822

[B34] Chun Y. S. Ryu K. Y. Ko Y. O. Hong J. Y. Hong J. Shin H. (2009). One-pot Synthesis of 2-pyridones via Chemo- and Regioselective Tandem Blaise Reaction of Nitriles with Propiolates. J. Org. Chem. 74, 7556–7558. 10.1021/jo901642t 19778084

[B35] Clercq E. D. (2004). Antivirals and Antiviral Strategies. Nat. Rev. Microbiol. 2, 704–720. 10.1038/nrmicro975 15372081 PMC7097272

[B36] Corte J. R. Fang T. Pinto D. J. P. Han W. Hu Z. Jiang X.-J. (2008). Structure-activity Relationships of Anthranilamide-Based Factor Xa Inhibitors Containing Piperidinone and Pyridinone P4 Moieties. Bioorg. Med. Chem. Lett. 18, 2845–2849. 10.1016/j.bmcl.2008.03.092 18424044

[B37] Cortes-Altamirano J. L. Olmos-Hernandez A. Jaime H. B. Carrillo-Mora P. Bandala C. Reyes-Long S. (2018). Review: 5-HT1, 5-HT2, 5-HT3 and 5-HT7 Receptors and Their Role in the Modulation of Pain Response in the Central Nervous System. Cn 16, 210–221. 10.2174/1570159x15666170911121027 PMC5883380 28901281

[B38] Crawford J. J. Johnson A. R. Misner D. L. Belmont L. D. Castanedo G. Choy R. (2018). Discovery of GDC-0853: A Potent, Selective, and Noncovalent Bruton's Tyrosine Kinase Inhibitor in Early Clinical Development. J. Med. Chem. 61, 2227–2245. 10.1021/acs.jmedchem.7b01712 29457982

[B39] Credille C. V. Chen Y. Cohen S. M. (2016). Fragment-Based Identification of Influenza Endonuclease Inhibitors. J. Med. Chem. 59, 6444–6454. 10.1021/acs.jmedchem.6b00628 27291165 PMC4948595

[B40] Crocetti L. Vergelli C. Guerrini G. Cantini N. Kirpotina L. N. Schepetkin I. A. (2020). Novel Formyl Peptide Receptor (FPR) Agonists with Pyridinone and Pyrimidindione Scaffolds that Are Potentially Useful for the Treatment of Rheumatoid Arthritis. Bioorg. Chem. 100, 103880. 10.1016/j.bioorg.2020.103880 32388428 PMC7409366

[B41] Daar E. S. (2008). Emerging Resistance Profiles of Newly Approved Antiretroviral Drugs. Top. HIV Med. 16, 110–116. 18838744

[B42] Daeyaert F. de Jonge M. Heeres J. Koymans L. Lewi P. Vinkers M. H. (2004). A Pharmacophore Docking Algorithm and its Application to the Cross-Docking of 18 HIV-NNRTI's in Their Binding Pockets. Proteins 54, 526–533. 10.1002/prot.10599 14748000

[B43] De Clercq E. (2001). New Developments in Anti-HIV Chemotherapy. Il Farmaco 56, 3–12. 10.1016/s0014-827x(01)01007-2 11347962

[B44] De Cock K. M. Jaffe H. W. Curran J. W. (2012). The Evolving Epidemiology of HIV/AIDS. Aids 26, 1205–1213. 10.1097/QAD.0b013e328354622a 22706007

[B45] Debnath U. Verma S. Jain S. Katti S. B. Prabhakar Y. S. (2013). Pyridones as NNRTIs against HIV-1 Mutants: 3D-QSAR and Protein Informatics. J. Comput. Aided Mol. Des. 27, 637–654. 10.1007/s10822-013-9667-1 23884707

[B46] Dias A. Bouvier D. Crépin T. McCarthy A. A. Hart D. J. Baudin F. (2009). The Cap-Snatching Endonuclease of Influenza Virus Polymerase Resides in the PA Subunit. Nature 458, 914–918. 10.1038/nature07745 19194459

[B47] Dingar D. Merlen C. Grandy S. Gillis M.-A. Villeneuve L. R. Mamarbachi A. M. (2010). Effect of Pressure Overload-Induced Hypertrophy on the Expression and Localization of P38 MAP Kinase Isoforms in the Mouse Heart. Cell Signal. 22, 1634–1644. 10.1016/j.cellsig.2010.06.002 20600854 PMC5298901

[B48] Dörr M. Meggers E. (2014). Metal Complexes as Structural Templates for Targeting Proteins. Curr. Opin. Chem. Biol. 19, 76–81. 10.1016/j.cbpa.2014.01.005 24561508

[B49] Drew W. L. (2010). Cytomegalovirus Resistance Testing: Pitfalls and Problems for the Clinician. Clin. Infect. Dis. 50, 733–736. 10.1086/650463 20100090

[B50] Engers J. L. Bollinger K. A. Weiner R. L. Rodriguez A. L. Long M. F. Breiner M. M. (2017). Design and Synthesis of N-Aryl Phenoxyethoxy Pyridinones as Highly Selective and CNS Penetrant mGlu3 NAMs. ACS Med. Chem. Lett. 8, 925–930. 10.1021/acsmedchemlett.7b00249 28947938 PMC5601378

[B51] Fedorov O. Castex J. Tallant C. Owen D. R. Martin S. Aldeghi M. (2015). Selective Targeting of the BRG/PB1 Bromodomains Impairs Embryonic and Trophoblast Stem Cell Maintenance. Sci. Adv. 1, e1500723. 10.1126/sciadv.1500723 26702435 PMC4681344

[B52] Fisher N. Meunier B. Biagini G. A. (2020). The Cytochromebc1complex as an Antipathogenic Target. FEBS Lett. 594, 2935–2952. 10.1002/1873-3468.13868 32573760

[B53] Getlik M. Smil D. Zepeda-Velázquez C. Bolshan Y. Poda G. Wu H. (2016). Structure-Based Optimization of a Small Molecule Antagonist of the Interaction between WD Repeat-Containing Protein 5 (WDR5) and Mixed-Lineage Leukemia 1 (MLL1). J. Med. Chem. 59, 2478–2496. 10.1021/acs.jmedchem.5b01630 26958703

[B54] Guillemont J. Benjahad A. Oumouch S. Decrane L. Palandjian P. Vernier D. (2009). Synthesis and Biological Evaluation of C-5 Methyl Substituted 4-arylthio and 4-Aryloxy-3-Iodopyridin-2(1h)-One Type Anti-HIV Agents. J. Med. Chem. 52, 7473–7487. 10.1021/jm900802y 19645483

[B55] Hambley T. W. (2007). Developing New Metal-Based Therapeutics: Challenges and Opportunities. Dalton Trans., 4929–4937. 10.1039/b706075k 17992277

[B56] Harada K. Ferdous T. Harada T. Takenawa T. Ueyama Y. (2017). Gimeracil Enhances the Antitumor Effect of Cisplatin in Oral Squamous Cell Carcinoma Cells *In Vitro* and *In Vivo* . Oncol. Lett. 14, 3349–3356. 10.3892/ol.2017.6602 28927087 PMC5587992

[B57] Hare S. Gupta S. S. Valkov E. Engelman A. Cherepanov P. (2010). Retroviral Intasome Assembly and Inhibition of DNA Strand Transfer. Nature 464, 232–236. 10.1038/nature08784 20118915 PMC2837123

[B58] Hartinger C. G. Dyson P. J. (2009). Bioorganometallic Chemistry-From Teaching Paradigms to Medicinal Applications. Chem. Soc. Rev. 38, 391–401. 10.1039/b707077m 19169456

[B59] He Y. She H. Zhang T. Xu H. Cheng L. Yepes M. (2018). p38 MAPK Inhibits Autophagy and Promotes Microglial Inflammatory Responses by Phosphorylating ULK1. J. Cel Biol 217, 315–328. 10.1083/jcb.201701049 PMC5748971 29196462

[B60] Hewings D. S. Rooney T. P. C. Jennings L. E. Hay D. A. Schofield C. J. Brennan P. E. (2012). Progress in the Development and Application of Small Molecule Inhibitors of Bromodomain-Acetyl-Lysine Interactions. J. Med. Chem. 55, 9393–9413. 10.1021/jm300915b 22924434

[B61] Hibi S. Ueno K. Nagato S. Kawano K. Ito K. Norimine Y. (2012). Discovery of 2-(2-Oxo-1-Phenyl-5-Pyridin-2-Yl-1,2-Dihydropyridin-3-Yl)benzonitrile (Perampanel): A Novel, Noncompetitive α-Amino-3-hydroxy-5-methyl-4-isoxazolepropanoic Acid (AMPA) Receptor Antagonist. J. Med. Chem. 55, 10584–10600. 10.1021/jm301268u 23181587

[B62] Hodson R. (2018). Alzheimer's Disease. Nature 559, S1. 10.1038/d41586-018-05717-6 30046078

[B63] Hohmann A. F. Martin L. J. Minder J. Roe J.-S. Shi J. Steurer S. (2016). Abstract LB-206: A Bromodomain-Swap Allele Demonstrates that On-Target Chemical Inhibition of BRD9 Limits the Proliferation of Acute Myeloid Leukemia Cells. Cancer Res. 76. LB-206-LB-206. 10.1158/1538-7445.AM2016-LB-206

[B64] Jacinto Demuner A. Moreira Valente V. M. Almeida Barbosa L. C. Rathi A. Donohoe T. Thompson A. (2009). Synthesis and Phytotoxic Activity of New Pyridones Derived from 4-Hydroxy-6-Methylpyridin-2(1h)-One. Molecules 14, 4973–4986. 10.3390/molecules14124973 20032871 PMC6255104

[B65] Jacobsen J. A. Fullagar J. L. Miller M. T. Cohen S. M. (2011). Identifying Chelators for Metalloprotein Inhibitors Using a Fragment-Based Approach. J. Med. Chem. 54, 591–602. 10.1021/jm101266s 21189019 PMC3024453

[B66] Jamieson E. R. Lippard S. J. (1999). Structure, Recognition, and Processing of Cisplatin−DNA Adducts. Chem. Rev. 99, 2467–2498. 10.1021/cr980421n 11749487

[B67] Javaid Z. Z. el Kouni M. H. Iltzsch M. H. (1999). Pyrimidine Nucleobase Ligands of Orotate Phosphoribosyltransferase from Toxoplasma Gondii. Biochem. Pharmacol. 58, 1457–1466. 10.1016/s0006-2952(99)00231-2 10513989

[B68] Jemal A. Siegel R. Xu J. Q. Ward E. (2013). Cancer Statistics. Cancer 52, 1–24. 10.1002/caac.20073

[B69] Jensen T. S. Finnerup N. B. (2014). Allodynia and Hyperalgesia in Neuropathic Pain: Clinical Manifestations and Mechanisms. Lancet Neurol. 13, 924–935. 10.1016/s1474-4422(14)70102-4 25142459

[B70] Jin J. Morales-Ramos Á. Eidam P. Mecom J. Li Y. Brooks C. (2010). Novel 3-Oxazolidinedione-6-Aryl-Pyridinones as Potent, Selective, and Orally Active EP3 Receptor Antagonists. ACS Med. Chem. Lett. 1, 316–320. 10.1021/ml100077x 24900213 PMC4007852

[B71] Johnson A. R. Kohli P. B. Katewa A. Gogol E. Belmont L. D. Choy R. (2016). Battling Btk Mutants with Noncovalent Inhibitors that Overcome Cys481 and Thr474 Mutations. ACS Chem. Biol. 11, 2897–2907. 10.1021/acschembio.6b00480 27571029

[B72] Karatas H. Townsend E. C. Cao F. Chen Y. Bernard D. Liu L. (2013). High-affinity, Small-Molecule Peptidomimetic Inhibitors of MLL1/WDR5 Protein-Protein Interaction. J. Am. Chem. Soc. 135, 669–682. 10.1021/ja306028q 23210835 PMC5180416

[B73] Kashuba A. D. Patterson K. B. Dumond J. B. Cohen M. S. (2012). Pre-exposure Prophylaxis for HIV Prevention: How to Predict success. The Lancet 379, 2409–2411. 10.1016/s0140-6736(11)61852-7 PMC3652584 22153566

[B74] Katrizky A. Ramsden C. A. Joule J. A. Zhdankin V. V. (2010). Handbook of Heterocyclic Chemistry. Third Edition, 41–42.

[B75] Kaufman J. DeLorenzo C. Choudhury S. Parsey R. V. (2016). The 5-HT1A Receptor in Major Depressive Disorder. Eur. Neuropsychopharmacol. 26, 397–410. 10.1016/j.euroneuro.2015.12.039 26851834 PMC5192019

[B76] Kim K. S. Zhang L. Schmidt R. Cai Z.-W. Wei D. Williams D. K. (2008). Discovery of Pyrrolopyridine−Pyridone Based Inhibitors of Met Kinase: Synthesis, X-ray Crystallographic Analysis, and Biological Activities. J. Med. Chem. 51, 5330–5341. 10.1021/jm800476q 18690676

[B77] Kranjc A. Mašič L. P. Reven S. Mikic K. Preželj A. Stegnar M. (2005). Novel Pyrazinone and Pyridinone Thrombin Inhibitors Incorporating Weakly Basic Heterobicyclic P1-Arginine Mimetics. Eur. J. Med. Chem. 40, 782–791. 10.1016/j.ejmech.2005.03.007 15890436

[B78] Lavanchy D. (2004). Hepatitis B Virus Epidemiology, Disease burden, Treatment, and Current and Emerging Prevention and Control Measures. J. Viral Hepat. 11, 97–107. 10.1046/j.1365-2893.2003.00487.x 14996343

[B79] Le Quesne J. P. Spriggs K. A. Bushell M. Willis A. E. (2010). Dysregulation of Protein Synthesis and Disease. J. Pathol. 220, 140–151. 10.1002/path.2627 19827082

[B80] Le Van K. Cauvin C. de Walque S. Georges B. Boland S. Martinelli V. (2009). New Pyridinone Derivatives as Potent HIV-1 Nonnucleoside Reverse Transcriptase Inhibitors. J. Med. Chem. 52, 3636–3643. 10.1021/jm801438e 19469474

[B81] León R. Garcia A. G. Marco-Contelles J. (2013). Recent Advances in the Multitarget-Directed Ligands Approach for the Treatment of Alzheimer's Disease. Med. Res. Rev. 33, 139–189. 10.1002/med.20248 21793014

[B82] Lin Z. G. Ren W. Zhang J. G. Miao P. (2003). Studies on Synthesis of 1-Amino-Y-Carboline Derivative. Chin. J. Med. Chem. 13, 267–269.

[B83] Liu J. Wang G.-H. Duan Y.-H. Dai Y. Bao Y. Hu M. (2017). Modulation of the Nur77-Bcl-2 Apoptotic Pathway by P38α MAPK. Oncotarget 8, 69731–69745. 10.18632/oncotarget.19227 29050237 PMC5642512

[B84] Liu Z. Yao Y. Kogiso M. Zheng B. Deng L. Qiu J. J. (2014). Inhibition of Cancer-Associated Mutant Isocitrate Dehydrogenases: Synthesis, Structure-Activity Relationship, and Selective Antitumor Activity. J. Med. Chem. 57, 8307–8318. 10.1021/jm500660f 25271760 PMC4207540

[B85] Louie A. Y. Meade T. J. (1999). Metal Complexes as Enzyme Inhibitors. Chem. Rev. 99, 2711–2734. 10.1021/cr9804285 11749498

[B86] Lubell Y. Dondorp A. Guérin P. J. Drake T. Meek S. Ashley E. (2014). Artemisinin Resistance - Modelling the Potential Human and Economic Costs. Malar. J. 13, 452. 10.1186/1475-2875-13-452 25418416 PMC4254187

[B87] Lv Z. Sheng C. Wang T. Zhang Y. Liu J. Feng J. (2010). Design, Synthesis, and Antihepatitis B Virus Activities of Novel 2-pyridone Derivatives. J. Med. Chem. 53, 660–668. 10.1021/jm901237x 20000776

[B88] Ma K. Zhang H. Baloch Z. (2016). Pathogenetic and Therapeutic Applications of Tumor Necrosis Factor-α (TNF-α) in Major Depressive Disorder: A Systematic Review. Ijms 17, 733. 10.3390/ijms17050733 PMC4881555 27187381

[B89] Maeda K. Das D. Kobayakawa T. Tamamura H. Takeuchi H. (2019). Discovery and Development of Anti-HIV Therapeutic Agents: Progress towards Improved HIV Medication. Ctmc 19, 1621–1649. 10.2174/1568026619666190712204603 PMC7132033 31424371

[B90] Martin L. J. Koegl M. Bader G. Cockcroft X.-L. Fedorov O. Fiegen D. (2016). Structure-Based Design of an *In Vivo* Active Selective BRD9 Inhibitor. J. Med. Chem. 59, 4462–4475. 10.1021/acs.jmedchem.5b01865 26914985 PMC4885110

[B91] Mbonye U. Karn J. (2014). Transcriptional Control of HIV Latency: Cellular Signaling Pathways, Epigenetics, Happenstance and the hope for a Cure. Virology 454-455, 328–339. 10.1016/j.virol.2014.02.008 24565118 PMC4010583

[B92] McElroy W. T. DeShong P. (2003). Siloxane-based Cross-Coupling of Bromopyridine Derivatives: Studies for the Synthesis of Streptonigrin and Lavendamycin. Org. Lett. 5, 4779–4782. 10.1021/ol0357503 14653672

[B93] Meggers E. (2009). Targeting Proteins with Metal Complexes. Chem. Commun., 1001–1010. 10.1039/b813568a 19225621

[B94] Melnikova I. (2009). The Anticoagulants Market. Nat. Rev. Drug Discov. 8, 353. 10.1038/nrd2851 19390569

[B95] Mendoza-Ferri M. G. Hartinger C. G. Mendoza M. A. Groessl M. Egger A. E. Eichinger R. E. (2009a). Transferring the Concept of Multinuclearity to Ruthenium Complexes for Improvement of Anticancer Activity. J. Med. Chem. 52, 916–925. 10.1021/jm8013234 19170599 PMC2819033

[B96] Mendoza-Ferri M. G. Hartinger C. G. Nazarov A. A. Eichinger R. E. Jakupec M. A. Severin K. (2009b). Influence of the Arene Ligand, the Number and Type of Metal Centers, and the Leaving Group on the *In Vitro* Antitumor Activity of Polynuclear Organometallic Compounds. Organometallics 28, 6260–6265. 10.1021/om900715j

[B97] Niewerth M. Kunze D. Seibold M. Schaller M. Korting H. C. Hube B. (2003). Ciclopirox Olamine Treatment Affects the Expression Pattern of Candida Albicans Genes Encoding Virulence Factors, Iron Metabolism Proteins, and Drug Resistance Factors. Antimicrob. Agents Chemother. 47, 1805–1817. 10.1128/AAC.47.6.1805-1817.2003 12760852 PMC155814

[B98] Niswender C. M. Conn P. J. (2010). Metabotropic Glutamate Receptors: Physiology, Pharmacology, and Disease. Annu. Rev. Pharmacol. Toxicol. 50, 295–322. 10.1146/annurev.pharmtox.011008.145533 20055706 PMC2904507

[B99] Novais Â. Moniz T. Rebelo A. R. Silva A. M. G. Rangel M. Peixe L. (2018). New Fluorescent Rosamine Chelator Showing Promising Antibacterial Activity against Gram-Positive Bacteria. Bioorg. Chem. 79, 341–349. 10.1016/j.bioorg.2018.05.013 29807207

[B100] Odobasic D. Jia Y. Kao W. Fan H. Wei X. Gu R. (2018). Formyl Peptide Receptor Activation Inhibits the Expansion of Effector T Cells and Synovial Fibroblasts and Attenuates Joint Injury in Models of Rheumatoid Arthritis. Int. Immunopharmacology 61, 140–149. 10.1016/j.intimp.2018.05.028 29879657

[B101] Pal Singh S. Dammeijer F. Hendriks R. W. (2018). Role of Bruton's Tyrosine Kinase in B Cells and Malignancies. Mol. Cancer 17, 57. 10.1186/s12943-018-0779-z 29455639 PMC5817726

[B102] Parhi A. K. Xiang A. Bauman J. D. Patel D. Vijayan R. S. K. Das K. (2013). Phenyl Substituted 3-Hydroxypyridin-2(1h)-Ones: Inhibitors of Influenza A Endonuclease. Bioorg. Med. Chem. 21, 6435–6446. 10.1016/j.bmc.2013.08.053 24055080

[B103] Paschka P. Schlenk R. F. Gaidzik V. I. Habdank M. Krönke J. Bullinger L. (2010). IDH1andIDH2Mutations Are Frequent Genetic Alterations in Acute Myeloid Leukemia and Confer Adverse Prognosis in Cytogenetically Normal Acute Myeloid Leukemia WithNPM1Mutation WithoutFLT3Internal Tandem Duplication. Jco 28, 3636–3643. 10.1200/jco.2010.28.3762 20567020

[B104] Pietrangelo T. Giampietro L. De Filippis B. La Rovere R. Fulle S. Amoroso R. (2010). Effect of Milrinone Analogues on Intracellular Calcium Increase in Single Living H9C2 Cardiac Cells. Eur. J. Med. Chem. 45, 4928–4933. 10.1016/j.ejmech.2010.08.001 20801556

[B105] Platts M. Y. Barber C. G. Chiva J.-Y. Eastwood R. L. Fenwick D. R. Paradowski K. A. (2011). A Concise Synthesis of HIV Integrase Inhibitors Bearing the Dipyridone Acid Motif. Tetrahedron Lett. 52, 512–514. 10.1016/j.tetlet.2010.11.112

[B106] Qin J.-J. Chen H.-X. Zhao N. Yuan L. Zhang Y.-Z. Yang R.-F. (2014). The Role of Activation of the 5-HT1A Receptor and Adenylate Cyclase in the Antidepressant-like Effect of YL-0919, a Dual 5-HT1A Agonist and Selective Serotonin Reuptake Inhibitor. Neurosci. Lett. 582, 104–108. 10.1016/j.neulet.2014.09.009 25220701

[B107] Robinson, M.D W. S. (1994). Molecular Events in the Pathogenesis of Hepadnavirus-Associated Hepatocellular Carcinoma. Annu. Rev. Med. 45, 297–323. 10.1146/annurev.med.45.1.297 8198385

[B108] Ruelas D. S. Greene W. C. (2013). An Integrated Overview of HIV-1 Latency. Cell 155, 519–529. 10.1016/j.cell.2013.09.044 24243012 PMC4361081

[B109] Sanchez R. I. Fillgrove K. L. Yee K. L. Liang Y. Lu B. Tatavarti A. (2019). Characterisation of the Absorption, Distribution, Metabolism, Excretion and Mass Balance of Doravirine, a Non-nucleoside Reverse Transcriptase Inhibitor in Humans. Xenobiotica 49, 422–432. 10.1080/00498254.2018.1451667 29557716

[B110] Santos R. Ursu O. Gaulton A. Bento A. P. Donadi R. S. Bologa C. G. (2017). A Comprehensive Map of Molecular Drug Targets. Nat. Rev. Drug Discov. 16, 19–34. 10.1038/nrd.2016.230 27910877 PMC6314433

[B111] Selness S. R. Boehm T. L. Walker J. K. Devadas B. Durley R. C. Kurumbail R. (2011). Design, Synthesis and Activity of a Potent, Selective Series of N -aryl Pyridinone Inhibitors of P38 Kinase. Bioorg. Med. Chem. Lett. 21, 4059–4065. 10.1016/j.bmcl.2011.04.120 21640588

[B112] Seo B. I. Uchil V. R. Okello M. Mishra S. Ma X.-H. Nishonov M. (2011). Discovery of a Potent HIV Integrase Inhibitor that Leads to a Prodrug with Significant Anti-HIV Activity. ACS Med. Chem. Lett. 2, 877–881. 10.1021/ml2001246 22328963 PMC3274775

[B113] Sheng R. Tang L. Jiang L. Hong L. Shi Y. Zhou N. (2016). Novel 1-Phenyl-3-Hydroxy-4-Pyridinone Derivatives as Multifunctional Agents for the Therapy of Alzheimer's Disease. ACS Chem. Neurosci. 7, 69–81. 10.1021/acschemneuro.5b00224 26479744

[B114] Shipley J. B. Tolman D. Hastillo A. Hess M. L. (1996). Milrinone: Basic and Clinical Pharmacology and Acute and Chronic Management. Am. J. Med. Sci. 311, 286–291. 10.1097/00000441-199606000-00011 8659556

[B115] Singh S. B. Liu W. Li X. Chen T. Shafiee A. Card D. (2012). Antifungal Spectrum, *In Vivo* Efficacy, and Structure-Activity Relationship of Ilicicolin H. ACS Med. Chem. Lett. 3, 814–817. 10.1021/ml300173e 24900384 PMC4025731

[B116] Slobodnyuk K. Radic N. Ivanova S. Llado A. Trempolec N. Zorzano A. (2019). Autophagy-induced Senescence Is Regulated by P38α Signaling. Cell Death Dis 10, 376. 10.1038/s41419-019-1607-0 31092814 PMC6520338

[B117] Smith S. J. Zhao X. Z. Passos D. O. Lyumkis D. Burke T. R. Jr. Hughes S. H. (2020). HIV-1 Integrase Inhibitors that Are Active against Drug-Resistant Integrase Mutants. Antimicrob. Agents Chemother. 64. 10.1128/aac.00611-20 PMC7449158 32601157

[B118] Song Y. n. Chen W. Kang D. Zhang Q. Zhan P. Liu X. (2014). "Old Friends in New Guise": Exploiting Privileged Structures for Scaffold Re-Evolution/Refining. Cchts 17, 536–553. 10.2174/1386207317666140122101631 24446784

[B119] Song Y. n. Zhan P. Liu X. (2013). Heterocycle-thioacetic Acid Motif: a Privileged Molecular Scaffold with Potent, Broad-Ranging Pharmacological Activities. Cpd 19, 7141–7154. 10.2174/13816128113199990505 23859548

[B120] Song Y. n. Zhan P. Zhang Q. Liu X. (2013). Privileged Scaffolds or Promiscuous Binders: a Glance of Pyrrolo[2,1-F][1,2,4]triazines and Related Bridgehead Nitrogen Heterocycles in Medicinal Chemistry. Cpd 19, 1528–1548. 10.2174/1381612811319080020 23131184

[B121] Stama M. L. Ślusarczyk J. Lacivita E. Kirpotina L. N. Schepetkin I. A. Chamera K. (2017). Novel Ureidopropanamide Based N-Formyl Peptide Receptor 2 (FPR2) Agonists with Potential Application for central Nervous System Disorders Characterized by Neuroinflammation. Eur. J. Med. Chem. 141, 703–720. 10.1016/j.ejmech.2017.09.023 29102463 PMC5693244

[B122] Stevaert A. Dallocchio R. Dessi A. Pala N. Rogolino D. Sechi M. (2013). Mutational Analysis of the Binding Pockets of the Diketo Acid Inhibitor L-742,001 in the Influenza Virus PA Endonuclease. J. Virol. 87, 10524–10538. 10.1128/jvi.00832-13 23824822 PMC3807387

[B123] Sweeney Z. K. Klumpp K. (2008). Improving Non-nucleoside Reverse Transcriptase Inhibitors for First-Line Treatment of HIV Infection: the Development Pipeline and Recent Clinical Data. Curr. Opin. Drug Discov. Devel 11, 458–470. 18600563

[B124] Temmink O. H. de Bruin M. Turksma A. W. Cricca S. Laan A. C. Peters G. J. (2007). Activity and Substrate Specificity of Pyrimidine Phosphorylases and Their Role in Fluoropyrimidine Sensitivity in colon Cancer Cell Lines. Int. J. Biochem. Cel Biol. 39, 565–575. 10.1016/j.biocel.2006.10.009 17098463

[B125] Thompson P. Manganiello V. Degerman E. (2007). Re-Discovering PDE3 Inhibitors - New Opportunities for a Long Neglected Target. Ctmc 7, 421–436. 10.2174/156802607779941224 17305583

[B126] Trono D. Van Lint C. Rouzioux C. Verdin E. Barré-Sinoussi F. Chun T.-W. (2010). HIV Persistence and the prospect of Long-Term Drug-free Remissions for HIV-Infected Individuals. Science 329, 174–180. 10.1126/science.1191047 20616270

[B127] Venkataraman S. Prasad B. Selvarajan R. (2018). RNA Dependent RNA Polymerases: Insights from Structure, Function and Evolution. Viruses 10, 76. Viruses 10. 10.3390/v10020076 PMC5850383 29439438

[B128] Vidler L. R. Brown N. Knapp S. Hoelder S. (2012). Druggability Analysis and Structural Classification of Bromodomain Acetyl-Lysine Binding Sites. J. Med. Chem. 55, 7346–7359. 10.1021/jm300346w 22788793 PMC3441041

[B129] Visseq A. Descheemaeker A. Pinto-Pardo N. Nauton L. Théry V. Giraud F. (2020). Pyridin-2(1H)one Derivatives: A Possible New Class of Therapeutics for Mechanical Allodynia. Eur. J. Med. Chem. 187, 111917. 10.1016/j.ejmech.2019.111917 31806536

[B130] Vrouenraets M. B. Visser G. W. Snow G. B. van Dongen G. A. (2003). Basic Principles, Applications in Oncology and Improved Selectivity of Photodynamic Therapy. Anticancer Res. 23, 505–522. 12680139

[B131] Wolańska M. Taudul E. Bańkowska-Guszczyn E. Kinalski M. (2010). Tumor Necrosis Factor in Uterine Leiomyomas at Various Stages of Tumor Growth. Ginekol Pol. 81, 431–434. 20695192

[B132] Workman D. G. Hunter M. Wang S. Brandel J. Hubscher V. Dover L. G. (2020). The Influence of Linkages between 1-Hydroxy-2(1h)-Pyridinone Coordinating Groups and a Tris(2-Aminoethyl)amine Core in a Novel Series of Synthetic Hexadentate Iron(III) Chelators on Antimicrobial Activity. Bioorg. Chem. 95, 103465. 10.1016/j.bioorg.2019.103465 31855824

[B133] Wu F. Jiang H. Zheng B. Kogiso M. Yao Y. Zhou C. (2015). Inhibition of Cancer-Associated Mutant Isocitrate Dehydrogenases by 2-Thiohydantoin Compounds. J. Med. Chem. 58, 6899–6908. 10.1021/acs.jmedchem.5b00684 26280302 PMC4567406

[B134] Wysocka J. Swigut T. Milne T. A. Dou Y. Zhang X. Burlingame A. L. (2005). WDR5 Associates with Histone H3 Methylated at K4 and Is Essential for H3 K4 Methylation and Vertebrate Development. Cell 121, 859–872. 10.1016/j.cell.2005.03.036 15960974

[B135] Xiao M. Li H. Wang Y. Wang X. Wang W. Peng J. (2006). Characterization of the N-Terminal Domain of Classical Swine Fever Virus RNA-dependent RNA Polymerase. J. Gen. Virol. 87, 347–356. 10.1099/vir.0.81385-0 16432021

[B136] Yan H. Parsons D. W. Jin G. McLendon R. Rasheed B. A. Yuan W. (2009). IDH1andIDH2Mutations in Gliomas. N. Engl. J. Med. 360, 765–773. 10.1056/NEJMoa0808710 19228619 PMC2820383

[B137] Yan Y. K. Melchart M. Habtemariam A. Sadler P. J. (2005). Organometallic Chemistry, Biology and Medicine: Ruthenium Arene Anticancer Complexes. Chem. Commun., 4764–4776. 10.1039/b508531b 16193110

[B138] Yeates C. L. Batchelor J. F. Capon E. C. Cheesman N. J. Fry M. Hudson A. T. (2008). Synthesis and Structure-Activity Relationships of 4-pyridones as Potential Antimalarials. J. Med. Chem. 51, 2845–2852. 10.1021/jm0705760 18396855

[B139] Young W. B. Barbosa J. Blomgren P. Bremer M. C. Crawford J. J. Dambach D. (2015). Potent and Selective Bruton's Tyrosine Kinase Inhibitors: Discovery of GDC-0834. Bioorg. Med. Chem. Lett. 25, 1333–1337. 10.1016/j.bmcl.2015.01.032 25701252

[B140] Yu M. Li P. K.C. Basnet S. Kumarasiri M. Diab S. Teo T. (2015). Discovery of 4-(dihydropyridinon-3-Yl)amino-5-Methylthieno[2,3-D]pyrimidine Derivatives as Potent Mnk Inhibitors: Synthesis, Structure-Activity Relationship Analysis and Biological Evaluation. Eur. J. Med. Chem. 95, 116–126. 10.1016/j.ejmech.2015.03.032 25800647

[B141] Zeng L. Zhou M.-M. (2002). Bromodomain: an Acetyl-Lysine Binding Domain. FEBS Lett. 513, 124–128. 10.1016/s0014-5793(01)03309-9 11911891

[B142] Zhang C. Yang K. Yu S. Su J. Yuan S. Han J. (2019). Design, Synthesis and Biological Evaluation of Hydroxypyridinone-Coumarin Hybrids as Multimodal Monoamine Oxidase B Inhibitors and Iron Chelates against Alzheimer's Disease. Eur. J. Med. Chem. 180, 367–382. 10.1016/j.ejmech.2019.07.031 31325784

[B143] Zhang Y. Pike A. (2021). Pyridones in Drug Discovery: Recent Advances. Bioorg. Med. Chem. Lett. 38, 127849. 10.1016/j.bmcl.2021.127849 33609656

[B144] Zhao X. Xin M. Huang W. Ren Y. Jin Q. Tang F. (2015). Design, Synthesis and Evaluation of Novel 5-Phenylpyridin-2(1h)-One Derivatives as Potent Reversible Bruton's Tyrosine Kinase Inhibitors. Bioorg. Med. Chem. 23, 348–364. 10.1016/j.bmc.2014.11.006 25515957

[B145] Zhou Z. Liu T. Zhang J. Zhan P. Liu X. (2018). Influenza A Virus Polymerase: an Attractive Target for Next-Generation Anti-influenza Therapeutics. Drug Discov. Today 23, 503–518. 10.1016/j.drudis.2018.01.028 29339107

[B146] Żmudzka E. Sałaciak K. Sapa J. Pytka K. (2018). Serotonin Receptors in Depression and Anxiety: Insights from Animal Studies. Life Sci. 210, 106–124. 10.1016/j.lfs.2018.08.050 30144453

